# Biased Estimates of Environmental Impact in the Negative Footprint Illusion: The Nature of Individual Variation

**DOI:** 10.3389/fpsyg.2021.648328

**Published:** 2022-01-18

**Authors:** Emma Threadgold, John E. Marsh, Mattias Holmgren, Hanna Andersson, Megan Nelson, Linden J. Ball

**Affiliations:** ^1^School of Psychology and Computer Science, University of Central Lancashire, Preston, United Kingdom; ^2^Engineering Psychology, Humans and Technology, Department of Business Administration, Technology and Social Sciences, Luleå University of Technology, Luleå, Sweden; ^3^Department of Building Engineering, Energy Systems, and Sustainability Science, University of Gävle, Gävle, Sweden; ^4^Department of Computer and Geospatial Sciences, University of Gävle, Gävle, Sweden; ^5^Department of Psychology, Lancaster University, Lancaster, United Kingdom

**Keywords:** negative footprint illusion, individual variation, reasoning, environment, actively open-minded thinking

## Abstract

People consistently act in ways that harm the environment, even when believing their actions are environmentally friendly. A case in point is a biased judgment termed the *negative footprint illusion*, which arises when people believe that the addition of “eco-friendly” items (e.g., environmentally certified houses) to conventional items (e.g., standard houses), *reduces* the total carbon footprint of the whole item-set, whereas the carbon footprint is, in fact, increased because eco-friendly items still contribute to the overall carbon footprint. Previous research suggests this illusion is the manifestation of an “averaging-bias.” We present two studies that explore whether people’s susceptibility to the negative footprint illusion is associated with individual differences in: (i) *environment-specific* reasoning dispositions measured in terms of compensatory green beliefs and environmental concerns; or (ii) *general* analytic reasoning dispositions measured in terms of actively open-minded thinking, avoidance of impulsivity and reflective reasoning (indexed using the Cognitive Reflection Test; CRT). A negative footprint illusion was demonstrated when participants rated the carbon footprint of conventional buildings combined with eco-friendly buildings (Study 1 and 2) and conventional cars combined with eco-friendly cars (Study 2). However, the illusion was not identified in participants’ ratings of the carbon footprint of apples (Study 1 and 2). In Studies 1 and 2, environment-specific dispositions were found to be unrelated to the negative footprint illusion. Regarding reflective thinking dispositions, reduced susceptibility to the negative footprint illusion was only associated with actively open-minded thinking measured on a 7-item scale (Study 1) and 17-item scale (Study 2). Our findings provide partial support for the existence of a negative footprint illusion and reveal a role of individual variation in reflective reasoning dispositions in accounting for a limited element of differential susceptibility to this illusion.

## Introduction

Climate change is one of the most significant challenges facing the modern world (Hansen et al., 2013). The Intergovernmental Panel on Climate Change (IPCC) estimates that anthropogenic greenhouse gas emissions will cause up to a 1.5 degrees centigrade global mean increase in surface air and sea temperature by approximately 2035 (Intergovernmental Panel on Climate Change [IPCC], 2020). Human activity, in the form of food production and general consumption, is directly associated with emissions of greenhouse gases (Carlsson-Kanyama, 1998) such as carbon dioxide. Whilst advances in technology have attempted to mitigate the impact of greenhouse gas emissions on the environment (Bradley, 2009), the most significant barrier to developing sustainability and mitigating climate change is psychological in nature (Gifford, 2011).

Evidence indicates that people consistently act in ways that harm the environment, even when they believe their actions to be environmentally friendly (e.g., Hope et al., 2018). It has been widely demonstrated that people incorrectly reason that the addition of “eco-friendly” (or “green”) items (e.g., environmentally certified houses) to a set of conventional items (e.g., standard houses) *reduces* the carbon footprint of the combined set of items (Holmgren et al., 2018a), whereas in fact the carbon footprint of the combined set *increases*. This reasoning bias is termed the *negative footprint illusion* (Gorissen and Weijters, 2016; Holmgren et al., 2018a,b; Sörqvist et al., 2020). The illusion has now been replicated many times, and has been shown to be insensitive to scale type (Gorissen and Weijters, 2016), expertise (Holmgren et al., 2018b), framing (Holmgren et al., 2019), quantity of additional items (i.e., “quantity insensitivity”; see Kim and Schuldt, 2018), experimental design (occurring in both between- and within-participants designs; see Holmgren et al., 2018a), and different stimulus materials such as foods (Gorissen and Weijters, 2016) and buildings (Holmgren et al., 2018a).

Research suggests that a cognitive bias referred to as “averaging bias” underpins the illusion, whereby reasoners fail to estimate the total environmental impact of a set of items, as requested, but instead provide judgments based on an assessment of the average environmental impact of items (Holmgren et al., 2018a). When environmentally friendly items are added to conventional items, an averaging process would readily give rise to the negative footprint illusion (Holmgren et al., 2018a). Consistent with research from Chernev and Gal (2010), people seem to engage in a “vice-virtue” categorization of items, whereby they classify different objects in a dichotomous fashion, for example, healthy versus unhealthy, or environmentally friendly versus conventional. After people have qualitatively classified objects, they subsequently produce a quantitative judgment, which is the average of their impact rather than their summative impact (Holmgren et al., 2019). This cognitive bias appears to be highly generalizable, with evidence indicating that it is present in a variety of real-world decision-making contexts, including in criminological (Lambert and Peytcheva, 2019), marketing (Weaver et al., 2012) and economic domains (Kunz et al., 2017).

Susceptibility to the negative footprint illusion can have an adverse impact on environment-related behavior and can plausibly exacerbate climate change. For example, people might buy more green products (which still have an environmental impact) than they normally would, in an effort to compensate for their use of conventional products (Sörqvist and Langeborg, 2019). It is, therefore, important to explore ways to eliminate the negative footprint illusion and its potential impact, which in turn necessitates acquiring a deeper understanding of the mechanisms that underpin the illusion and the nature of individual variation in people’s susceptibility to it. The aim of the present research was to undertake empirical studies that might shed further light on the role played by individual differences in people’s susceptibility to the negative footprint illusion.

### Dispositional Individual Differences and the Negative Footprint Illusion

Dispositional factors have previously been implicated in relation to people’s susceptibility to the negative footprint illusion, in terms of the extent to which individuals manifest “compensatory green beliefs” (Kaklamanou et al., 2015). Compensatory green beliefs reflect people’s disposition to believe that endorsing particular pro-environment behaviors (e.g., not driving a car) can serve to compensate for less environment-friendly behaviors (e.g., not recycling). Kaklamanou et al. (2015) found that the overall endorsement of compensatory green beliefs is relatively low, but is nevertheless negatively correlated with demographic and dispositional factors – with increasing age, a higher income and educational status all being related to a lower endorsement of compensatory green beliefs. Furthermore, greater endorsement of compensatory green beliefs was found to be negatively associated with pro-ecological behavior, as measured in terms of General Ecological Behaviour (GEB), a willingness to engage in pro-ecological behavior (Kaiser et al., 2003) and green identity (e.g., Whitmarsh and O’Neill, 2010). MacCutcheon et al. (2020) have recently shown that people who were more likely to endorse compensatory green beliefs are more susceptible to the negative footprint illusion compared to individuals with lower endorsement scores, and therefore this forms a potentially important element of individual variation in susceptibility to the illusion.

However, the illusion does not appear to be solely explained by individual variation in environment-related dispositions. Research suggests the illusion persists even when controlling for measures such as environmental concern (Gorissen and Weijters, 2016), ecological values (Kim and Schuldt, 2018) and green consumer values (Kusch and Fiebelkorn, 2019). Such findings raise doubts about the impact of environment-related dispositional factors in explaining susceptibility to the negative footprint illusion. Critically, there has been scarce consideration of individual variation in the illusion, arising from more general dispositional factors related to thinking and reasoning. According to Kabanshi (2020), our ability to reason about environmental impact is biased in systematic ways, and behaviors detrimental to the environment are believed to be linked to general, deep-seated cognitive and dispositional factors associated with reasoning, judgment and decision-making. It therefore seems plausible that individual variation in *general* reflective reasoning dispositions could shed further light on people’s tendency to implement an averaging process of the type that appears to underpin the negative footprint illusion. Such individual variation could, for example, take the form of the adoption of particular thinking styles (e.g., Stanovich, 2009) or tendencies toward impulsivity in responding (e.g., Moeller et al., 2001).

One thinking style or disposition that has been studied extensively is referred to as “actively open-minded thinking” (e.g., Sá et al., 1999; Stanovich and West, 2007). This captures individual variation in people’s motivation to engage in rational thought (Stanovich, 2009) and has been demonstrated to be independent of cognitive ability, but highly correlated with people’s success in making normatively rational judgments (Sá and Stanovich, 2001). Actively open-minded thinking has also been found to be related to the objective evaluation of arguments (Stanovich and West, 1998), alongside the ability to avoid falling foul of various types of cognitive bias, including confirmation bias (Baron, 1993) and knowledge bias (Sá and Stanovich, 2001). It is typically measured on a self-report scale, of which several versions have been constructed, each involving various compositions of latent factors (see Stanovich and West, 1997, 2007; Haran et al., 2013; Svedholm-Häkkinen and Lindeman, 2018). It is noteworthy that, to date, there has been no exploration of how generic thinking styles might be related to reduced susceptibility to the negative footprint illusion.

In exploring thinking styles, a critical issue relates to the extent to which individuals engage in “intuitive” versus “reflective” thought (e.g., Evans, 2010, 2018; Evans and Stanovich, 2013a,b). According to Evans and Stanovich (2013a,b), thinking can be categorized into two qualitatively distinct types of mental processes, that is, “Type 1,” intuitive, heuristic processes versus “Type 2,” reflective, analytic processes. According to this “dual process” perspective on reasoning, Type 1 processes have two defining features: (i) they are relatively undemanding of working memory resources; and (ii) they are autonomous, which means that they run obligatorily to completion whenever they are activated. Type 1 processes also tend to be fast, high capacity, non-conscious and capable of operating in parallel, but these are merely correlated rather than defining features. Type 2 processes, on the other hand, are defined in terms of requiring working memory resources (Evans, 2008) and being focused on cognitive decoupling and mental simulation, which are critical for determining the viability of default judgments arising from Type 1 processes. Type 2 processes also tend to be slow, capacity limited, conscious and serial, but again, these are viewed by Evans and Stanovich (2013a,b) as being correlated features rather than defining features.

From a dual-process perspective, actively open-minded thinking can be viewed as a measure of people’s disposition to engage in Type 2 reasoning, thereby potentially enabling biases to be overcome that have arisen from the operation of intuitive, Type 1 processing. This view, in which intuitive Type 1 processes provide *default* reasoning responses that can be reflected on and potentially overturned or accepted by Type 2 processes, is referred to as involving a “default-interventionist” processing structure (e.g., Evans and Stanovich, 2013a,b). It is unclear, however, whether the averaging bias that underpins the negative footprint illusion is a consequence of an autonomous, “rough-and-ready,” intuitive approach to averaging happening at a Type 1 level or is a result of more reflective Type 2 reasoning that is simply inappropriate for the task at hand, which requires a summative judgment.

As both Evans (2012, 2018) and Stanovich (2018) are at pains to point out, biased reasoning is not just the preserve of Type 1 processing, as biases can also arise during Type 2 processing, for example, through applying effective reasoning processes to a misconstrued problem representation or through the application of sub-optimal, reflective processes (i.e., “defective mindware”; Stanovich, 2018). One possible explanation for what is arising in the case of individuals who fall foul of the negative footprint illusion is that they are misconstruing the problem from the outset and applying an “intuitive” averaging process based on a qualitative “vice-virtue” categorization of items. If such individuals do go on to apply Type 2 reflective reasoning, then they will only overcome the averaging bias if they appreciate that the task requires a summative judgment as opposed to one based on averaging. If Type 2 reasoning does not elicit this realization (e.g., through a careful check of the instructions), then any further Type 2 processing that is applied to the task would simply serve to *rationalize* the averaging-based judgment arising from the default, Type 1 process. This account would predict that individuals higher in actively open-minded thinking dispositions might be more likely to appreciate from the outset that the task requires summation or, alternatively, that they might be more likely to engage in a checking process at the Type 2 stage, which would reveal that an averaging response is not what is required and that a summation response is needed. Either way, increased levels of actively open-minded thinking should be associated with reduced susceptibility to the negative footprint illusion.

Another task in the reasoning literature that is claimed to capture people’s dispositions to engage in reflective reasoning is the Cognitive Reflection Test (CRT; Kahneman and Frederick, 2002). This involves presenting participants with problems that lend themselves to relatively immediate and intuitive responses that seem correct, but which are, in fact, incorrect and need to be overturned by reflective thinking to attain the correct solution. Toplak et al. (2011) highlight that it is a particularly strong performance measure of the extent to which individuals can overcome intuitive errors that result from “miserly processing” by engaging in further reflective thought (but see Stupple et al., 2017, for a critical analysis of the role of miserly processing in CRT performance, and Stanovich, 2018, for counterarguments). Of relevance to the current research is the potential for the CRT to offer a measure of rational thought that is not otherwise explained by common variables such as executive functioning and intelligence, but which nevertheless captures the disposition for people to engage in reflective reasoning and their ability to do so effectively (i.e., the CRT can perhaps best be viewed as both a dispositional *and* an ability measure; Campitelli and Gerrans, 2014).

In sum, the present research aimed to advance an understanding of the role played by individual differences in people’s susceptibility to the negative footprint illusion, with a key focus being placed on environment-specific reasoning dispositions as well as dispositional factors relating to the engagement of Type 2, reflective processes that may promote more accurate reasoning.

## Study 1

In this study, we sought to determine the extent to which susceptibility to the negative footprint illusion is associated with: (i) environment-specific dispositions, measured by the endorsement of compensatory green beliefs and the use of the Environmental Concerns Questionnaire; (ii) general dispositions toward Type 2, analytic reasoning, measured in terms of an actively open-minded thinking (AOT) scale; and (iii) general dispositions toward, and abilities at, Type 2 reasoning, as measured by performance on the Cognitive Reflection Test (CRT).

We also included two further individual differences measures as exploratory variables. The first was a measure of “impulsivity” to capture impulsive personality traits (e.g., Barratt, 1959), which may play a role in incorrect responding on negative footprint illusion tasks. The second was a measure of people’s inclination to make incorrect probability judgments when reasoning with problems that give rise to the so-called “conjunction fallacy,” whereby people rate the conjunction of two events as being more likely than either event alone (Tversky and Kahneman, 1983; see also Fantino et al., 1997). We included this measure because it has previously been suggested that the illusion bears some conceptual similarity to fallacious reasoning about conjunctive events (Holmgren et al., 2018a). More specifically, both the conjunction fallacy and the negative footprint illusion appear to revolve around people making biased estimates regarding the conjunction of attributes or events, albeit assigning a higher value to a conjunctive probability in the case of the conjunction fallacy and assigning a lower value to a conjunctive carbon footprint in the case of the negative footprint illusion. Of course, the two biases may not necessarily be underpinned by the same cognitive operations or mechanisms (cf. Holmgren et al., 2018a), but their apparent conceptual overlap represents an issue worth investigating empirically. We finally note that this study afforded an opportunity to determine whether the well-documented negative footprint illusion holds in the case of small objects (i.e., apples) in addition to large items (i.e., buildings), which have featured more extensively in prior studies.

### Method

#### Participants

The participants were 120 adults (72 male) with a mean age of 36 years (*SD* = 13 years). Participants were recruited via Prolific Academic and received the standard platform payment rate. The study received Ethical Clearance from the University of Gävle, Sweden.

#### Design

A within-participants design with two factors was employed: item type (buildings vs. apples) and carbon footprint estimation task (conventional vs. conventional plus eco-friendly “green” addition). The dependent variables were the carbon footprint rating for each task and the outcomes of each of the individual difference measures (environmental concerns, endorsement of compensatory green beliefs, actively open-minded thinking, impulsiveness, CRT performance and conjunction fallacy susceptibility).

#### Materials

##### Carbon Footprint Estimation Tasks

###### Rating the Carbon Footprint of Apples

Participants were presented with contextual information pertaining to the carbon footprint of apples, and a graphic depicting 10 conventional apples in a consumer’s food basket. They were asked to make a judgment rating of the carbon footprint of these 10 apples on a scale from “low carbon footprint” (‘1’) to “high carbon footprint” (‘9’). Participants were then informed that the consumer had returned to the store and added five eco-friendly apples to their basket. Participants were then required to estimate the carbon footprint of the 15 apples in the consumer’s food basket on a scale from “low carbon footprint” (‘1’) to “high carbon footprint” (‘9’).

###### Rating the Carbon Footprint of Buildings

Participants were asked to provide a rating for the number of trees required to compensate for the carbon footprint arising from the energy used by a community of buildings (i.e., houses). Participants were presented with contextual information, alongside a graphic of 75 conventional buildings marked in orange, and information relating to greenhouse gas emissions. Participants were asked to mark the number of trees used to compensate for energy use on a scale from 1 to 100. They were then presented with the same contextual information as previously, alongside a description of the additional 25 green buildings. A graphic depicting 75 conventional buildings in orange together with the 25 new eco-friendly buildings in green was presented. They were asked once again to mark on the scale from 1 to 100 how many trees they estimated the suburb would need to compensate for its energy use per month. Please see the Supplementary Material for detailed carbon footprint estimation task descriptions and graphics.

##### Environment Specific Dispositional Measures

###### Biospheric, Altruistic and Egoistic Environmental Concerns Questionnaire

The Biospheric, Altruistic and Egoistic Environmental Concerns Questionnaire (see Schultz, 2001) involves 12 items, with participants being asked to rate each item on a 9-point scale (1 = not at all concerned to 9 = very concerned) in response to questions that are framed as follows: “*How concerned are you that today’s environmental problems will affect.*?” The 12 presented items related to three areas of environmental concern: biospheric (e.g., animals), altruistic (e.g., future generations) and egoistic (e.g., my future).

###### Compensatory Green Beliefs Questionnaire

Green beliefs were measured using a 16-item questionnaire (see Kaklamanou et al., 2015). Participants were asked to rate on a 5-point scale (1 = strongly disagree to 5 = strongly agree) how closely each statement fitted their beliefs (e.g., *“Not driving a car compensates for not recycling”*).

##### General Dispositional Measures of Reasoning

###### Actively Open-Minded Thinking Scale

The study utilized the shortened 7-item version of the Actively Open-Minded Thinking Scale (AOT-7), developed by Haran et al. (2013). Participants were asked to respond to seven statements (e.g., *“Allowing oneself to be convinced by an opposing argument is a sign of good character”*) on a 7-point scale, ranging from 1 (completely disagree) to 7 (completely agree).

###### Cognitive Reflection Test

Participants were presented with the 6-item CRT developed by Primi et al. (2016) and referred to by them as the CRT-L (for CRT-Long). The 6-item version contains three items originally used by Frederick (2005) and a further three items added by Primi et al. (2016) to create the CRT-L. Please refer to the Supplementary Material for details of the CRT-L and the scoring method.

##### Other Measures

###### Barratt Impulsiveness Scale

The Barratt Impulsiveness Scale (Barratt, 1959; Patton et al., 1995; Stanford et al., 2009) is a 30-item questionnaire designed to measure impulsive personality traits in terms of the ways in which participants think and act (e.g., *“I am happy-go-lucky”*). Each item is rated on a 4-point scale (1 = rarely/never, 2 = occasionally, 3 = often, 4 = almost always/always).

###### Susceptibility to the Conjunction Fallacy

To assess people’s susceptibility to the conjunction fallacy two tasks were utilized from the materials presented by Tversky and Kahneman (1983): the famous “Linda Problem” and a less well known “Die-Roll Problem.” Participants were asked to select their answer from the options provided. Please refer to the Supplementary Material for details of these tasks and the scoring method.

#### Procedure

The study was deployed using Qualtrics. Participants read an information sheet and completed a consent form. A carbon footprint rating task (apples or buildings item) was completed, with item type counterbalanced across participants. For each task, participants were presented with instructions and contextual information. They were asked to make their initial carbon footprint rating for the set of conventional items, followed by a second rating for the conventional items with the addition of the eco-friendly items. On completing each rating, participants were asked to indicate on a 9-point scale, the extent to which they solved the problem via either “intuition” (‘1’) or “analysis” (‘9’). Please refer to the Supplementary Material for definitions of these terms. Participants were also asked to respond on a scale from “not at all confident” (‘1’) to “very confident” (‘9’) to indicate their confidence in their given response for each of the carbon footprint rating tasks.

On completion of one of the two carbon footprint rating judgments, participants then completed the individual differences questionnaires in a fixed order, as follows: (i) Biospheric, Altruistic and Egoistic Environmental Concerns; (ii) AOT-7; (iii) Barratt Impulsiveness Scale; and (iv) Compensatory Green Beliefs Questionnaire. The CRT-L questions were then presented in a random order. Participants were given a maximum time of 3 min per question to generate their solution and write their response in the text field. The two conjunction fallacy problems were then presented, with participants permitted up to 3 min to respond to each problem from the fixed response options provided. Participants were then presented with the second carbon footprint rating task (apples or buildings), with the initial carbon footprint rating with conventional items being followed by a second rating with the addition of eco-friendly items. Finally, participants were debriefed.

### Results

#### Rating the Carbon Footprint for the Apples and Buildings Item Types

A 2 × 2 repeated-measures analysis of variance (ANOVA) test was conducted on the carbon footprint ratings (mean proportions) to identify the presence of a negative footprint illusion. This revealed a significant main effect of item type on carbon footprint ratings, *F*(1,119) = 55.29, *p* < 0.001, $\eta_{\text{p}}^{2}$ = 0.32, with carbon footprint ratings being significantly higher for the buildings item type (*M* = 0.66, *SE* = 0.02) than for the apples item type (*M* = 0.46, *SE* = 0.02). There was no significant main effect of the carbon footprint estimation task, *F*(1,119) = 0.49, *p* = 0.484, $\eta_{\text{p}}^{2}$ = 0.01, with ratings for the conventional item estimate (*M* = 0.56, *SE* = 0.01) being similar to ratings for the conventional plus eco-friendly items estimate (*M* = 0.55, *SE* = 0.02).

There was a significant interaction between item type and carbon footprint estimation task, *F*(1,119) = 14.60, *p* < 0.001, $\eta_{\text{p}}^{2}$ = 0.11. Pairwise comparisons with a Bonferroni adjustment revealed the presence of a negative footprint illusion in the case of the buildings item type (*p* = 0.004), with estimates for the conventional buildings (*M* = 0.68, *SE* = 0.02) significantly higher than estimates for the conventional plus eco-friendly buildings (*M* = 0.64, *SE* = 0.03). However, there was no such negative footprint illusion for the apples item type, with ratings for conventional apples (*M* = 0.44, *SE* = 0.02) being significantly *lower* (*p* = 0.027) than for the ratings for conventional apples with the addition of eco-friendly apples (*M* = 0.47, *SE* = 0.02).

#### Ratings of Solution Confidence for the Apples and Buildings Item Types

A 2 × 2 repeated measures ANOVA revealed that participants were significantly more confident in their judgments of the carbon footprint of the apples item type (*M* = 4.15, *SE* = 0.17) than in their judgments for the buildings item type (*M* = 2.84, *SE* = 0.15), *F*(1,119) = 62.92, *p* < 0.001, $\eta_{\text{p}}^{2}$ = 0.35, seemingly indicating a degree of accurate metacognitive awareness given that greater confidence with the apples item type was also associated with normatively correct responding in relation to the carbon footprint estimation task for this item type. In contrast, less confidence arose for carbon footprint estimates associated with the buildings item type, which also gave rise to negative footprint illusion.

There was no significant main effect of carbon footprint estimation task in terms of whether the rating was for conventional items alone (*M* = 3.55, *SE* = 0.14) in comparison to conventional items with the addition of eco-friendly items (*M* = 3.44, *SE* = 3.44), *F*(1,119) = 2.46, *p* = 0.119, $\eta_{\text{p}}^{2}$ = 0.02, although the means indicate that participants were slightly less confident in providing their second rating of the conventional plus eco-friendly items combined. There was no significant interaction between item type and carbon footprint estimation task for confidence ratings, *F*(1,119) = 0.77, *p* = 0.384, $\eta_{\text{p}}^{2}$ = 0.01.

#### Ratings of Solution Strategies for the Apples and Buildings Item Types

A 2 × 2 repeated measures ANOVA revealed that solutions strategies were self-reported to be significantly more intuitive in the case of carbon footprint ratings for the buildings item type (*M* = 3.37, *SE* = 0.16) in comparison to the apples item type (*M* = 4.45, *SE* = 0.18), *F*(1,119) = 32.42, *p* < 0.001, $\eta_{\text{p}}^{2}$ = 0.01. There was no significant main effect of carbon footprint estimation task, *F*(1,119) = 1.02, *p* = 0.315, $\eta_{\text{p}}^{2}$ = 0.01, with self-reported solution strategies for the first estimate (*M* = 4.45, *SE* = 0.18) not being significantly different to self-reported solution strategies for the second estimate (*M* = 3.37, *SE* = 0.16), although the mean values indicate that the second estimates were slightly more intuitive than the more analytic first estimates. There was no significant interaction between item type and carbon footprint estimation task, *F*(1,119) = 0.54, *p* = 0.465, $\eta_{\text{p}}^{2}$ = 0.01. These solution-strategy findings again suggest a degree of metacognitive awareness on the part of participants, who seem to be able to sense that their responses to the buildings item type (which gave rise to a negative footprint illusion) are more intuitive than their responses to the apples item type (which gave rise to normative responding).

#### Individual Differences Measures

The Biospheric, Altruistic and Egoistic Environmental Concerns Questionnaire, Compensatory Green Beliefs Questionnaire, Barratt Impulsiveness Scale (with subdivision into three second order factors; attentional, motor and non-planning impulsiveness) and the AOT-7 were all scored according to instructions, with higher scores reflecting a greater degree of each trait. The CRT-L was scored according to the process outlined by Pennycook et al. (2016). A CRT-*Reflective* score was derived to measure the ability of individuals to overcome intuitive responses and reach a normatively correct response, whilst the CRT-*Intuitive* score was derived representing a measure of solutions that spring to mind rapidly and seem plausible, but which are incorrect.

To provide a measure of an individual’s susceptibility to the negative footprint illusion, a score was calculated for each item type (apples or buildings) to indicate the extent of *change* between the estimate for conventional items only and the second estimate for conventional plus eco-friendly items. The raw score for the conventional items only was subtracted from the raw score for the conventional plus eco-friendly items, resulting in either a 0 (no change), a positive score (no negative footprint illusion) or a negative score (a negative footprint illusion) for each participant. These change scores formed the key measure of susceptibility to the negative footprint illusion that was then used in computing correlations with individual differences measures. Table 1 shows Pearson correlation coefficients for the relationship between the change scores for the carbon footprint ratings for both the apples and buildings item types and all measures of individual differences: environmental concerns, endorsement of compensatory green beliefs, impulsiveness, actively open-minded thinking, CRT-*Reflective* and CRT-*Intuitive*. Skewness and kurtosis scores were computed for each variable to determine whether scores approached a normal distribution and were deemed to fall within an acceptable range (Bulmer, 2003).

**TABLE 1:** Correlation matrix showing the relationship (Pearson correlation coefficients) between individual differences measures and the carbon footprint change scores for the apples and buildings item types.

Variables	1.	2.	3.	4.	5.	6.	7.	8.	9.	10.	11.
1. Change score for apples item type											
2. Change score for buildings item type	0.059										
3. Environmental concerns	0.062	−0.015									
4. Compensatory green beliefs	0.111	−0.057	−0.224*								
5. Impulsivity	0.007	−0.106	−0.195*	0.327**							
6. Attentional impulsivity	0.092	−0.132	−0.167	0.340**	0.797**						
7. Motor impulsivity	−0.091	0.023	−0.030	0.162	0.765**	0.416**					
8. Non-planning impulsivity	0.029	−0.148	−0.264**	0.298**	0.851**	0.583**	0.423**				
9. Actively open-minded thinking	0.107	0.186*	0.119	−0.344**	−0.424**	−0.242**	−0.375**	−0.387**			
10. CRT-*Intuitive*	−0.028	−0.065	0.118	0.141	0.250**	0.156	0.257**	0.187*	−0.202*		
11. CRT-*Reflective*	0.055	0.106	−0.204*	−0.130	−0.208*	−0.103	−0.243**	−0.149	0.221*	−0.865**	

*N = 120 for each cell.*

*Correlation is significant at the p < 0.05.

**Correlation is significant at the p < 0.001.

The key finding from Table 1 is a significant positive correlation between actively open-minded thinking and the change score for the buildings item type (*r* = 0.186, *p* = 0.042, with 3.5% of the variance in the change score accounted for). Given that a positive change score is indicative of a normatively correct response to the carbon footprint estimation task, this correlation suggests that greater actively open-minded thinking is associated with a *reduction* in susceptibility to the negative footprint illusion for the buildings item type. The conjunction fallacy tasks were scored by noting a correct or an incorrect response for each participant for both the Linda Problem and the Die-Roll Problem. Point bi-serial correlations indicated there was no relationship between the ability to endorse the likelihood of one single event occurring (more so than two events happening in conjunction) and reduced susceptibility to the negative footprint illusion (all *p*s > 0.05).

### Discussion

The findings of this study support previous research in partially replicating a negative footprint illusion (Gorissen and Weijters, 2016; Holmgren et al., 2018a,b). Estimating the number of trees necessary to offset the carbon footprint of a community of conventional buildings, along with a community of additional eco-friendly buildings, was judged to require fewer trees than a community comprised of conventional buildings alone. In contrast, no negative footprint illusion was demonstrated in the context of rating the carbon footprint of apples (participants correctly judged that a basket of 10 conventional apples and 5 eco-friendly apples would have a greater carbon footprint than a basket of 10 conventional apples alone).

Interestingly, participants’ confidence ratings and strategy ratings in relation to their carbon footprint estimates indicated the presence of a metacognitive component, with confidence ratings and analytic reasoning ratings being significantly higher where there was no susceptibility to the illusion (apples task), than where susceptibility to the illusion was present (buildings task). These findings may provide some converging evidence in support of the negative footprint illusion being underpinned by Type 1 reasoning, as would arise from the operation of an intuitively applied averaging bias. We do note, however, the need for caution in this interpretation of the metacognitive data, given that the instructional context surrounding the buildings task was more complex than that surrounding the apples task. Therefore, the metacognitive judgments were potentially sensitized to the perceived differential complexity of the buildings and apples tasks, rather than to differences in the underpinning reasoning processes used to generate carbon footprint estimates.

Study 1 provides little evidence in support of an influence of either general or more environment-specific thinking dispositions in susceptibility to the negative footprint illusion. With respect to environment-specific thinking dispositions, the findings failed to reveal any effect of environmental concern (Schultz, 2001) or the endorsement of compensatory green beliefs (Kaklamanou et al., 2015) in explaining susceptibility to the negative footprint illusion. This lack of evidence is at odds with previous findings reported by MacCutcheon et al. (2020), which demonstrate a link between compensatory green beliefs and susceptibility to the negative footprint illusion. We note, however, that MacCutcheon et al. (2020) demonstrated this significant association using a 9-point scale, rather than the 5-point scale adopted in the present study, which suggests that our replication failure might be attributable to a lack of sensitivity arising from a reduced range of scale.

A variety of dispositional factors that relate to general reflective reasoning ability were also explored, including actively open-minded thinking, performance on the cognitive reflection test and the conjunction fallacy and impulsivity. These questionnaires and tasks failed to predict susceptibility to the negative footprint illusion, with the single exception being that of actively open-minded thinking, as measured by the AOT-7 scale (Haran et al., 2013). Higher scores on this scale significantly predicted reduced susceptibility to the illusion on the buildings item type, albeit with a somewhat small degree of variance (3.5%). However, we note that the AOT-7 may have the potential to lead to spurious findings because of limitations in its capacity to measure actively open-minded thinking in an accurate manner, with questions being raised recently regarding its validity and reliability (e.g., Svedholm-Häkkinen and Lindeman, 2018).

## Study 2

In Study 2, we sought to replicate and extend the findings of Study 1 whilst also increasing the test power of the study. Study 1 revealed a negative footprint illusion with buildings as the carbon producing item, yet it failed to identify the illusion in the apples task. This finding is at odds with previous research demonstrating the presence of a negative footprint illusion across various types of items, including foods (Gorissen and Weijters, 2016), buildings (Holmgren et al., 2018a), and cars (Holmgren et al., 2021). We suggest that there are three potential explanations for this absence of a negative footprint illusion with the apples task.

First, the illusion might be less pronounced for small physical items (e.g., fruit), in comparison to larger items (e.g., cars or buildings). Second, it might be sensitive to the relative quantity of eco-friendly “green” items that are added to conventional items. In this respect we note that Study 1 implemented tasks with 75 conventional houses plus 25 eco-friendly houses (i.e., the eco-friendly addition was 33% of the conventional item set) versus 10 conventional apples plus 5 eco-friendly apples (i.e., the eco-friendly addition was 50% of the item set). Third, the scale that was employed with the apples task, where ratings were possible in the range from 1 to 9, might have led to the absence of a negative footprint illusion because this scale was less sensitive than the one used in the context of the buildings task, which ranged from 1 to 100.

In Study 2, we sought to disentangle these competing explanations by implementing three key changes to the carbon footprint estimation tasks. First, we included an additional carbon footprint estimation with an item between the size of apples and buildings. In this task participants were asked to rate the carbon footprint of a fleet of conventional cars, followed by a rating for the same fleet of conventional cars to which eco-friendly cars had been added. Second, the carbon footprint estimation scale was made consistent across each of the three tasks to avoid any impact of scale sensitivity. Third, the ratio of conventional items to eco-friendly additions was standardized across each task, resulting in eco-friendly additions equal to 50% of the conventional items (e.g., 10 apples plus 5 eco-friendly “green” apples).

As in Study 1, we aimed to determine the extent to which susceptibility to the negative footprint illusion is associated with environment-specific dispositions (measured by the endorsement of compensatory green beliefs and the environmental concerns). However, in a deviation from Study 1, the response scale for the Compensatory Green Beliefs Questionnaire was extended from a 5-point to a 9-point Likert scale. The purpose of this was twofold: (i) to increase the sensitivity to the scale; and (ii) to maintain consistency with previous research (MacCutcheon et al., 2020), which revealed a significant relationship between endorsement of compensatory green beliefs and the negative footprint illusion.

The current study also sought once again to explore the relationship between people’s general dispositions toward Type 2, reflective reasoning and the negative footprint illusion, but with some amendments to the scales and tasks. Study 1 employed the brief AOT-7 scale (Haran et al., 2013) as opposed to the full AOT-41 scale (e.g., Stanovich and West, 1997; Sá et al., 1999). The AOT-7 correlates relatively poorly with the AOT-41 (*r* = 0.66) in comparison to the recently developed AOT-17 scale (*r* = 0.89; Svedholm-Häkkinen and Lindeman, 2018). Furthermore, the AOT-7 does not permit the exploration of the latent factors underpinning the scale (dogmatism, fact resistance, liberalism and belief personification). Therefore, in Study 2 we employed the AOT-17, which retains an acceptable level of internal consistency (see Heijltjes et al., 2015), provides a stronger correlation with the original AOT-41, and permits an examination of the latent sub-factors.

In Study 2, we also employed a shortened version of the CRT, adopting only the three items that were added to the original version by Primi et al. (2016). We implemented this change as we were concerned about the familiarity that our research participants had with the original three item CRT, especially given suggestions that prior exposure to the CRT might result in higher scores (Haigh, 2016). Study 1 indicated that participants’ familiarity of the original CRT items (i.e., whether they had previously encountered any of the items) was much greater (33%) than for the new items of the CRT-L (0.03%). That said, comparable mean percentage solution rates were observed between the original CRT (45%) and the CRT-L (50%), which aligns with Bialek and Pennycook’s (2018) evidence that prior exposure to the CRT does not influence its predictive power. Nevertheless, to mitigate against any issues relating to prior exposure to the original CRT, we decided simply to employ the new three items from the CRT-L.

Study 1 failed to show any association between incorrect judgments in the conjunction fallacy tasks and individual differences in susceptibility to the negative footprint illusion and we therefore omitted these tasks from Study 2. However, we retained the measure of impulsivity (Barratt, 1959) to capture any potential impulsive personality traits that might play a role in incorrect responding on tasks that induce a negative footprint illusion. In this respect it is noteworthy that in Study 1 the impulsivity scale showed a highly significant positive correlation with the CRT-*Intuitive* measure as well as highly significant negative correlation with the CRT-*Reflective* measure and AOT score, suggesting overlapping variance with these measures that might manifest in individual differences in people’s susceptibility to the negative footprint illusion in a higher-powered study.

### Method

#### Participants

A power analysis was employed to determine an appropriate *a priori* sample size. This indicated a minimum sample size of 266 participants for a 0.4 Cohen’s *d* with 90% power. The participants were 269 adults (93 male) with a mean age of 31 years (*SD* = 11 years). All participants were recruited via Prolific Academic and received the standard platform payment. The study received Ethical Clearance from the Ethics Board (PsySoc: 507) at the University of Central Lancashire, United Kingdom.

#### Design

A 3 × 2 within-participants design was employed. The factors were item type (buildings vs. cars vs. apples) and carbon footprint estimation task (conventional vs. conventional plus eco-friendly “green” addition). The dependent variables were the carbon footprint rating for each task and the outcomes of each of the individual difference measures (environmental concerns, endorsement of compensatory green beliefs, actively open-minded thinking, impulsiveness, CRT performance), which are described in more detail below. As in Study 1, additional measures were also taken for self-reported solution strategies for the carbon footprint estimation tasks and subjective confidence in solutions.

#### Materials

##### Carbon Footprint Estimation Tasks

Three carbon footprint estimation tasks were constructed, in which participants were asked to rate the carbon footprint of apples, cars and buildings. Participants were given contextual information relating to both the conventional items, and the conventional + “green” eco-friendly items. Participants rated the carbon footprint of a basket of 10 apples, and subsequently the carbon footprint of a basket of 10 apples + 5 “green” eco-friendly apples (15 apples in total). They rated the carbon footprint of a fleet of 30 petrol cars, and subsequently the carbon footprint of a fleet of 30 petrol cars + 15 “green” eco-friendly cars (45 cars in total). Finally, they rated the carbon footprint of a community of 50 conventional houses, and a community of 50 conventional houses + 25 “green” eco-friendly houses (75 houses in total).

For each of the three tasks, participants made their conventional and conventional plus “green” eco-friendly addition carbon footprint estimation ratings on a sliding scale from 1 (1ow carbon footprint) to 100 (high carbon footprint). The score attributed to the scale position was not visible to participants when making their estimation. This was to minimize participants’ attempts to make carbon footprint estimations relative to a previous task scenario. The sliding scale was set to the middle (this translated to a score of 50). In contrast to Study 1, participants were provided with an anchor point for each task, relative to each carbon producing item. For example, in the context of the apples task participants were provided with the following: “*As a reference point for your estimations, consider that a basket of 10 mixed fruit would score in the middle of the scale.”* Further details of each negative footprint illusion task can be found in the Supplementary Material. Following each carbon footprint estimation, participants rated their confidence in their given response (on a scale from 1 = “not very confident” to 9 = “very confident’) and indicated their solution strategy (on a scale from 1 = “intuition” to 9 = “analysis”). Please see the Supplementary Material for detailed carbon footprint estimation task descriptions and graphics.

##### Environmental and General Dispositional (Individual Differences) Measures

These measures remained largely consistent with Study 1, with the following notable changes. First, in the compensatory green beliefs task, participants rated each statement on a 9-point scale (1 = “strongly disagree” to 9 = “strongly agree”), rather than on a 5-point scale, as in Study 1. Second, the conjunction fallacy tasks were omitted. Third, the AOT-7 was replaced with the AOT-17 (Svedholm-Häkkinen and Lindeman, 2018). Finally, we retained just the three less familiar items from the 6-item CRT-L (Primi et al., 2016).

#### Procedure

Participants read an information sheet and completed a check box consent form. They completed one of the three carbon footprint estimation tasks, provided a confidence and solution strategy rating, before completing the individual differences questionnaires in a fixed order: (i) Biospheric, Altruistic and Egoistic Environmental Concerns; (ii) AOT-17; (iii) Barratt Impulsiveness Scale; and (iv) Compensatory Green Beliefs Questionnaire. Following the presentation of the four scales, participants completed a further carbon footprint estimation task, followed by the three CRT questions, presented in a random order. Participants then completed the final carbon footprint estimation task. Carbon footprint estimation task presentation order was fully counterbalanced across participants. Participants were debriefed at the end of the study.

### Results

#### Rating the Carbon Footprint for the Apples, Cars and Buildings Item Types

Analyses were conducted on the raw carbon footprint estimation scores (1 indicating a low carbon footprint to 100 indicating a high carbon footprint). A 3 (item type: apples vs. cars vs. buildings) × 2 (rating: conventional vs. conventional plus eco-friendly “green”) repeated-measures ANOVA was conducted on the carbon footprint ratings to explore the presence of a negative footprint illusion across the three different carbon producing item types. A Mauchley’s Test of Sphericity indicated that the assumption of sphericity had been violated for both item type (χ*^2^* = 48.94, *p* < 0.001) and the rating by item type interaction (χ*^2^* = 33.15, *p* < 0.001) and a Greenhouse–Geisser correction was applied.

There was a significant main effect of item type, *F*(1.71,459.10) = 539.92, *p* < 0.001, $\eta_{\text{p}}^{2}$ = 0.67. The carbon footprint ratings were significantly higher for the cars item type (*M* = 73.84, *SE* = 0.91) in comparison to the buildings item type (*M* = 69.28, *SE* = 0.89, *p* < 0.001) or the apples item type (*M* = 36.66, *SE* = 1.03, *p* < 0.001). The carbon footprint ratings for the buildings item type were significantly higher than for the apples item type (*p* < 0.001). Incidentally, participants estimated cars to have the highest carbon footprint, followed by buildings, and then apples. The ratings for the conventional items only (*M* = 63.51, *SE* = 0.62) were significantly higher than for the conventional plus eco-friendly “green” items (*M* = 56.34, *SE* = 0.81); *F*(1,268) = 97.02, *p* < 0.001, $\eta_{\text{p}}^{2}$ = 0.27. A significant reduction in rating scores with the addition of eco-friendly “green” items, indicates the presence of a general negative footprint illusion.

There was a significant interaction between item type and rating, *F*(1.79,479.95) = 50.26, *p* < 0.001, $\eta_{\text{p}}^{2}$ = 0.16. Pairwise comparisons with a Bonferroni adjustment applied for multiple comparisons were conducted to reveal the source of the interaction. A negative footprint illusion was identified for the buildings item type, with the conventional plus eco-friendly “green” ratings (*M* = 64.06, *SE* = 1.13) resulting in significantly lower carbon footprint estimations than for the conventional ratings only (*M* = 74.49, *SE* = 0.89), *p* < 0.001. This effect was also found with the cars item type, again with conventional plus eco-friendly “green” ratings (*M* = 68.50, *SE* = 1.15) resulting in significantly lower carbon footprint estimations than for the conventional ratings only (*M* = 79.17, *SE* = 0.97), *p* < 0.001. However, for the apples item type, the conventional ratings (*M* = 36.86, *SE* = 1.12) did not differ significantly from the conventional plus eco-friendly “green” ratings (*M* = 36.46, *SE* = 1.12), *p* = *0.65*, therefore suggesting the presence of a “zero footprint illusion” for this task (see Holmgren et al., 2021).

#### Ratings of Solution Confidence for the Apples, Cars and Buildings Item Types

A further 3 (item type: apples vs. cars vs. buildings) × 2 (rating: conventional vs. conventional plus eco-friendly “green”) repeated-measures ANOVA was conducted on the raw confidence rating scores. A Mauchley’s Test of Sphericity indicated that the assumption of sphericity had been violated for both item type (χ*^2^* = 17.38, *p* < 0.001) and the rating by item type interaction (χ*^2^* = 7.18, *p* = 0.028). A Greenhouse–Geisser correction was applied to adjust for the violation of sphericity. A main effect of item type was identified, *F*(1.88,504.23) = 27.79, *p* < 0.001, $\eta_{\text{p}}^{2}$ = 0.09. Participants indicated significantly higher confidence when rating the carbon footprint of cars (*M* = 5.71, *SE* = 0.09) in comparison to buildings (*M* = 5.40, *SE* = 0.10) and apples (*M* = 5.00, *SE* = 0.11), both *p*s < 0.001. Participants were also significantly more confident when rating the carbon footprint of buildings in comparison to apples (*p* < 0.001). Furthermore, a main effect of rating, *F*(1,268) = 11.39, *p* < 0.001, $\eta_{\text{p}}^{2}$ = 0.04, indicated that confidence was greatest when making a carbon footprint rating of conventional items (*M* = 5.46, *SE* = 0.09) in comparison to conventional plus “green” eco-friendly items (*M* = 5.28, *SE* = 0.09).

There was a significant interaction between item type and rating, *F*(1.95,522.14) = 5.99, *p* = 0.003, $\eta_{\text{p}}^{2}$ = 0.02. Pairwise comparisons with a Bonferroni adjustment explored the source of this interaction. For the buildings task, there was no significant difference in confidence ratings for the conventional rating (*M* = 5.44, *SE* = 0.11) and the conventional plus eco-friendly rating (*M* = 5.36, *SE* = 0.10), *p* = 0.30. The same pattern was found for the apples task; there was no significant difference in confidence ratings for the conventional rating (*M* = 5.03, *SE* = 0.12) and the conventional plus eco-friendly rating (*M* = 4.98, *SE* = 0.11), *p* = 0.60. However, for the cars task, participants were significantly more confident in their conventional rating (*M* = 5.91, *SE* = 0.10) in comparison to their conventional plus eco-friendly rating (*M* = 5.51, *SE* = 0.10), *p* < 0.001.

#### Ratings of Solution Strategy for the Apples, Cars and Buildings Item Types

A final 3 (item type: apples vs. cars vs. buildings) × 2 (rating: conventional vs. conventional plus eco-friendly “green”) repeated-measures ANOVA was conducted on the raw solution strategy rating scores. A Greenhouse–Geisser correction was applied to the rating by item type interaction effect (χ^2^ = 17.24, *p* < 0.001), given a Mauchley’s Test of Sphericity revealed the assumption of sphericity had been violated. For the solution strategy ratings, there was a significant main effect of item type, *F*(2,536) = 12.03, *p* < 0.001, $\eta_{\text{p}}^{2}$ = 0.04, with no significant difference in solution strategy between the buildings item type (*M* = 5.02, *SE* = 0.11) and cars item type (*M* = 5.03, *SE* = 0.12), *p* = 1.00. Solution strategy responses were significantly more intuitive for the apples item type (*M* = 4.56, *SE* = 0.11) in comparison to the buildings and cars item types (both *p*s < 0.001). There was a significant main effect of rating, *F*(1,268) = 4.00, *p* = 0.047, $\eta_{\text{p}}^{2}$ = 0.02. Conventional ratings were significantly more intuitive (*M* = 4.80, *SE* = 0.10) than conventional plus eco-friendly ratings (*M* = 4.91, *SE* = 0.10). For the solution strategy scores, there was no significant interaction between item type and rating, *F*(1.82,504.46) = 1.01, *p* = 0.33, $\eta_{\text{p}}^{2}$ = 0.00.

#### Individual Differences Measures

Consistent with Study 1, a change score was computed for the apples, cars and buildings tasks. For each of the change scores, and the individual differences variables, scores were checked for skew and kurtosis, and all were deemed to be within an acceptable range. The relationship (Pearson correlation coefficients) between the change scores for the apples, cars and buildings tasks and individual difference measures (environmental concerns, compensatory green beliefs, impulsivity, CRT-*Reflective* and CRT-*Intuitive*) are shown in Table 2. The relationship between the change scores and the AOT-17 total score and sub-scales (dogmatism, fact resistance, liberalism and belief personification) and impulsivity, are shown in Table 3.

**TABLE 2:** Correlation matrix showing the relationship (Pearson correlation coefficients) between individual differences measures and the carbon footprint change scores for the apples, cars and buildings item types.

Variables	1.	2.	3.	4.	5.	6.	7.	8.	9.
1. Change score for apples item type									
2. Change score for cars item type	0.135*								
3. Change score for buildings item type	0.113	0.591**							
4. Environmental concerns	0.053	−0.071	−0.031						
5. Compensatory green beliefs	0.086	−0.097	−0.011	−0.210**					
6. Impulsivity	−0.087	−0.036	−0.094	0.003	0.111				
7. Attentional impulsivity	0.055	0.034	−0.010	0.112	0.036	0.595**			
8. Motor impulsivity	−0.126*	−0.059	−0.114	0.047	0.105	0.823**	0.261**		
9. Non-planning impulsivity	−0.082	−0.034	−0.069	−0.106	0.099	0.852	0.359**	0.512**	

*N = 269 for each cell.*

*Correlation is significant at the p < 0.05.

**correlation is significant at the p < 0.001.

**TABLE 3:** Correlation matrix showing the relationship (Pearson correlation coefficients) between the actively open-minded thinking scale and sub-scales (dogmatism, fact resistance, liberalism and belief personification), impulsivity and the carbon footprint change scores for the apples, cars and buildings item types.

Variables	1.	2.	3.	4.	5.	6.	7.	8.	9.	10.	11.
1. Change score for apples item type											
2. Change score for cars item type	0.135*										
3. Change score for buildings item type	0.113	0.591**									
4. Actively open-minded thinking (AOT)	0.099	0.226**	0.143*								
5. AOT dogmatism	0.081	0.205**	0.082	0.875**							
6. AOT fact resistance	0.106	0.201**	0.200**	0.816**	0.613**						
7. AOT liberalism	−0.071	0.035	0.012	0.407**	0.253**	0.268**					
8. AOT belief personification	0.083	0.097	0.043	0.448**	0.218**	0.097	−0.062				
9. CRT-*Intuitive*	0.074	−0.037	−0.053	−0.061	−0.116	−0.059	0.067	0.032			
10. CRT-*Reflective*	−0.074	0.104	0.118	0.212**	0.228**	0.160**	−0.005	0.099	−0.723*		
11. Impulsivity	−0.087	−0.036	−0.094	−0.077	−0.033	−0.062	0.049	−0.137*	0.115	−0.101	

*N = 269 for each cell.*

*Correlation is significant at the p < 0.05.

**Correlation is significant at the p < 0.001.

Table 2 reveals that environmental concerns, compensatory green beliefs and impulsivity are not associated with the change scores for the apples, cars or buildings tasks. Table 3 reveals that the overall AOT-17 score is significantly positively correlated with the change scores for the buildings task (*r* = 0.143, *p* = 0.019, with 2% of the variance in the change score being accounted for), and the cars task (*r* = 0.226, *p* < 0.001, with 5.1% of the variance in the change score accounted for), but not for the apples task (*r* = 0.099, *p* = 0.106), for which no negative footprint illusion was found. Thus, the disposition to engage in Type 2 actively open-minded thinking is associated with a significant reduction in susceptibility to the negative footprint illusion in the context of both the buildings and cars item types. Table 3 also indicates that the change score for the buildings task was significantly related to the fact resistance sub-scale of the AOT-17 (*r* = 0.200, *p* < 0.001), whilst the change scores for the cars tasks was significantly related to both the fact resistance (*r* = 0.201, *p* < 0.001) and dogmatism sub-scales (*r* = 0.205, *p* < 0.001). Some protection from susceptibility to the negative footprint illusion therefore seems to be afforded by the ability to engage in actively open-minded thinking, particularly in terms of fact resistance, and potentially with respect to dogmatic thinking.

It is also important to note that borderline significant correlations were obtained between the ability to engage in reflective thought (as measured by the CRT-*Reflective* score) and the buildings task change score (*r* = 0.111, *p* = 0.053) and the cars task change score (*r* = 0.104, *p* = 0.088). Thus, there is some evidence to indicate that the ability to engage in Type 2 reflective thinking (indexed by the CRT) might offer protection from susceptibility to the negative footprint illusion.

### Discussion

The findings of this study confirmed the presence of a significant negative footprint illusion for participants rating the carbon footprint of larger carbon producing objects in the form of cars (cf. Holmgren et al., 2021) and buildings (cf. Holmgren et al., 2018a), when: (i) the carbon footprint estimation was measured on a consistent scale across these carbon producing item types; (ii) a relative anchor point was provided for each conventional (initial) carbon footprint estimate; and (iii) the ratio of conventional versus conventional plus eco-friendly items was kept constant across item types. A reduction of approximately 10% in participants’ ratings of the combined carbon footprint of conventional plus eco-friendly (“green”) buildings or cars was observed over the carbon footprint of a set of conventional buildings or cars on their own.

In terms of the apples task, we observed a zero footprint illusion, whereby participants did not appear to attribute any significant increase *or* decrease in their carbon footprint estimation. The occurrence of a zero footprint illusion has previously been seen in the literature (Holmgren et al., 2021) and can be viewed as also reflecting non-normative performance, although the mechanism that underpins the manifestation of this effect remains unclear. The absence of a negative footprint illusion with the apples task is contrary to previous research that has demonstrated the illusion with other items of food (e.g., Gorissen and Weijters, 2016). We speculate about potential reasons for the absence of the illusion with the apples task in the “General Discussion” section.

The confidence ratings for the carbon footprint estimations indicate a degree of metacognitive awareness in responding, as was also evident in Study 1. For the cars task, participants were significantly more confident in their conventional only carbon footprint estimations, in comparison to their conventional plus eco-friendly estimations. The solution strategy ratings where a negative footprint illusion was observed (i.e., for the cars and buildings tasks), were significantly more analytic in nature, than for the apples task, where more intuitive responses were noted. Furthermore, the conventional ratings were deemed by participants to be significantly more intuitive thanthe conventional plus eco-friendly ratings. We note, however, that the pattern of findings relating to confidence and strategy judgments is different to that seen in Study 1, which suggests that these measures may be highly context-sensitive for reasons that we consider further in the “General Discussion” section.

In terms of the role of environment-specific reasoning dispositions and their relation to individual variation in susceptibility to the negative footprint illusion, the findings from this study remain consistent with those from Study 1. Study 2 did not demonstrate any significant relationship with either environmental concerns (Schultz, 2001), or endorsement of compensatory green beliefs (Kaklamanou et al., 2015). The role of environment-specific reasoning has been disputed in the literature on the negative footprint illusion, with only one study to date demonstrating a relationship between endorsement of compensatory green beliefs and the number of trees required to offset the carbon footprint of a community of buildings (MacCutcheon et al., 2020), with this relationship yet to be replicated.

Although the findings from the present study did not indicate any role for environment-specific reasoning dispositions in the manifestation of the negative footprint illusion, our findings instead provide evidence for a limited role of *general* dispositions to engage in Type 2 reflective reasoning. These dispositions were measured in this study in terms of actively open-minded thinking, as indexed by the AOT-17, which showed a strong correlation with the avoidance of the negative footprint illusion on the buildings and cars tasks. These Type 2 reflective reasoning dispositions were also indexed by CRT-*Reflective* scores, which showed a weak but nevertheless non-significant correlation with avoidance of the negative footprint illusion – again for the buildings and cars tasks. Measures of Type 1 intuitive responding in terms of impulsivity (Barratt, 1959) or as indexed by the CRT-*Intuitive* scores showed no reliable associations with the emergence of a negative footprint illusion on any of the tasks.

We note that the use of the 17-item AOT scale (Svedholm-Häkkinen and Lindeman, 2018) in the present study supported the findings revealed in Study 1 with the shorter 7-item AOT scale (Haran et al., 2013). The propensity to engage in actively open-minded thinking was associated with a reduced susceptibility to the negative footprint illusion when measured on the longer and more reliable 17-item scale. It remains debatable as to whether actively open-minded thinking is a unitary phenomenon, and Svedholm-Häkkinen and Lindeman (2018) have argued for the presence of four distinct yet intercorrelated sub-factors. An exploration of these sub-factors revealed a key role of reduced fact-resistance and reduced dogmatic thinking in underpinning the significant relationship between reduced susceptibility to the negative footprint illusion and actively open-minded thinking. In addition, we note the significant correlation identified between the engagement in reflective thought (CRT-*Reflective*), and the AOT-17 (*r* = 0.212, *p* < 0.001), which provides a further demonstration of the overlapping relationship between these measures, supporting the view that actively open-minded thinking aligns closely with the disposition to engage in Type 2 reflective thinking (e.g., Stanovich, 2009).

## General Discussion

We have presented two empirical studies in which we demonstrated a negative footprint illusion in environmental decision making. In Study 1, the illusion was observed when participants were required to estimate the number of trees that would need to be planted to offset the carbon footprint of a community of conventional and eco-friendly “green” buildings. In Study 2, we replicated this finding when participants were asked to rate the carbon footprint of conventional and eco-friendly “green” buildings. Furthermore, we revealed in Study 2 that the carbon footprint illusion generalizes to rating the carbon footprint of conventional (petrol) cars and eco-friendly (electric) cars (cf. Holmgren et al., 2018a). These studies provide clear-cut evidence for a negative footprint illusion. However, it is also apparent that under some circumstances, participants can, in fact, engage in normative responding. More specifically, participants did not fall foul of the negative footprint illusion in the context of determining the carbon footprint of conventional and eco-friendly apples in Study 1. In Study 2, participants provided similar ratings for the conventional apples versus the conventional plus eco-friendly apples, thus demonstrating the presence of a zero footprint illusion whereby neither a significant increase *nor* decrease in carbon footprint ratings was demonstrated. Our findings therefore provide support for the existence of a negative footprint illusion in the context of rating the carbon footprint of buildings and cars, but not in the context of rating the carbon footprint of apples.

The normative responding in the context of the apples task in Study 1 and the presence of a zero footprint illusion with the apples task in Study 2, is at odds with previous research that has demonstrated the extension of the illusion to food items in the form of burgers (Kusch and Fiebelkorn, 2019), although not to fruit specifically. Our findings therefore indicate a potential role for the *volume* of CO_2_ output in driving the emergence of a negative footprint illusion. We also note that although we did not explicitly require participants to make carbon footprint ratings of objects relative to each other, it is possible that participants displayed some sensitivity to object size as a consequence of our implementation of a counterbalanced repeated-measures design whereby *repeated* carbon footprint estimations were made. In Study 2, we observed significantly higher carbon footprint ratings for the cars and buildings tasks, in comparison to the apples task, despite retaining a consistent scale and a relative anchor point for each task.

We note, however, that not only did the apples task in Study 2 contain the smallest CO_2_ producing items in the form of small fruit in comparison to either cars or buildings, but it also contained the lowest total number of objects across the three item types (i.e., 75 houses vs. 45 cars vs. 15 apples). This gives rise to the possibility that the limited “numerosity” of conventional items and eco-friendly additions might play a role in the absence of a negative footprint illusion with the apples task. In other words, whilst we standardized the *relative* number of conventional and eco-friendly items across tasks, the *absolute* number of items in the apples task was much lower than in the other two tasks. This numerosity issue might reduce the salience of the eco-friendly additions and diminish the averaging bias that is assumed to underpin task performance. This speculative hypothesis clearly requires further systematic investigation in future studies. We additionally explored participants’ relative confidence in responding as well as the self-reported solution strategies that they attributed to each carbon footprint estimation across each of the tasks. Interestingly, in Study 1, participants’ confidence ratings and strategy ratings in relation to their carbon footprint estimates indicated the presence of a metacognitive component to these estimates, with confidence ratings and analytic reasoning ratings being significantly higher in the task involving apples, where there was no susceptibility to the negative footprint illusion, than in the task involving buildings, where susceptibility to the illusion was present. These metacognitive findings may provide some converging evidence in support of the negative footprint illusion being underpinned by Type 1 reasoning, as would arise from the operation of an intuitively applied averaging bias.

However, we note a word of caution in the interpretation of these latter metacognitive findings. The instructional context surrounding the buildings task in Study 1 was more complex than that surrounding the apples task. It might therefore be that participants’ metacognitive judgments were sensitized to the perceived differential complexity of the buildings and apples item types, rather than to differences in the underpinning reasoning processes used to generate carbon footprint estimates. Indeed, this explanation is somewhat supported by the findings of Study 2, in which we simplified and streamlined the contextual information for each of the three tasks. It was then noted in Study 2, that participants’ confidence in responding was highest in the tasks where they were susceptible to the illusion (the buildings and cars tasks) in comparison to the apples task, where a zero footprint illusion was identified. However, the confidence ratings were significantly higher for the conventional only ratings, than for the conventional plus eco-friendly ratings, perhaps suggesting some degree of awareness of the more cognitively challenging nature of the second conventional plus eco-friendly rating.

In terms of solution strategy, participants in Study 2 rated their responses as significantly more intuitive in the apples task in comparison to the buildings and cars tasks, whilst conventional ratings were significantly more intuitive than conventional plus eco-friendly ratings. These findings do not provide support for the concept of metacognitive awareness indicating a role of Type 1 intuitive reasoning in susceptibility to the negative footprint illusion. However, it is important to note that in Study 2, most of the solution strategy ratings fall around the middle of the 9-point response scales, with relatively low standard error values. Whilst participants were required to move the slider to prevent mid-scale responding, the findings do indicate a tendency for responding around the middle of the intuition versus analysis continuum scale, implying that participants were not making a commitment to responding in terms of either an intuitive or an analytic solution strategy.

The key focus of the present research was on the nature of individual differences in susceptibility to the negative footprint illusion. With respect to environment-specific thinking dispositions, our findings failed to reveal any effect of environmental concern (Schultz, 2001) or of the endorsement of compensatory green beliefs (Kaklamanou et al., 2015) in explaining susceptibility to the negative footprint illusion. This lack of evidence is at odds with previous findings reported by MacCutcheon et al. (2020), which demonstrated a link between compensatory green beliefs and susceptibility to the negative footprint illusion. Whilst we note methodological differences in the negative footprint illusion tasks employed here, thus preventing an exact replication, we failed to identify any such relationship when using both a 5-point scale (Study 1), and a 9-point scale (Study 2), with the latter being consistent with that employed by MacCutcheon et al. (2020).

The present research also explored a variety of measures that are indicative of a disposition to engage in Type 2 reflective thinking. These measures included actively open-minded thinking and performance on the CRT. We also investigated measures that are arguably indicative of a more intuitive, Type 1 thinking style, that is, impulsivity and susceptibility to the conjunction fallacy. In Study 1, nearly all these questionnaires and tasks failed to predict susceptibility to the negative footprint illusion, with the single exception being that of the actively open-minded thinking scale (AOT-7) when predicting susceptibility to the negative footprint illusion with the buildings item type, but not with the apples item type. Study 2 further supported this latter finding, with evidence that actively open-minded thinking was again associated with reduced susceptibility to the negative footprint illusion in the context of buildings and cars, but similarly to Study 1, not apples. In Study 1, the AOT-7 scale (Haran et al., 2013) predicted reduced susceptibility to the illusion on the buildings item type. Study 2 employed the longer AOT-17 scale (Svedholm-Häkkinen and Lindeman, 2018). The AOT-17 significantly predicted reduced susceptibility to the illusion in the context of both the buildings item type and the cars item type. These findings are the first that we are aware of to suggest that an increase in the disposition to engage in Type 2 actively open-minded thinking is associated with a limited degree of reduced vulnerability to the negative footprint illusion in the context of buildings and cars item types.

Actively open-minded thinking has previously been found to be positively associated with the belief that human activity is responsible for global warming (e.g., Stenhouse et al., 2018) and climate change (e.g., Kahan and Corbin, 2016). Such findings raise the possibility that individual beliefs regarding the anthropogenic causes of climate change might themselves be potentially important in predicting people’s reduced susceptibility to the negative footprint illusion. Furthermore, actively open-minded thinking is associated with the acceptance of counterintuitive ideas (Sinatra et al., 2003) and the ability to evaluate arguments objectively (Stanovich and West, 1997). It is also associated with decreased vulnerability to cognitive biases such as belief bias (West et al., 2008) and confirmation bias (Baron, 1993). These prior findings align with the present observations that actively open-minded thinking reduces susceptibility to the negative footprint illusion, presumably because participants who score highly on the AOT-7 and AOT-17 scales are able to avoid succumbing to the averaging bias that has been claimed to form the underpinning basis of the illusion (e.g., Holmgren et al., 2018a).

The AOT-17 scale in Study 2 additionally permitted an exploration of the role of latent sub-factors (Svedholm-Häkkinen and Lindeman, 2018). This exploration revealed that the sub-factors associated with the carbon footprint estimation change scores in the cars task were dogmatism and fact resistance, whilst the only sub-factor associated with the carbon footprint estimation change scores in the buildings task was fact resistance. Dogmatism refers to the belief that there is a single, correct philosophy or way of doing things that people should follow. Fact resistance refers to one’s tendency never to abandon beliefs, regardless of the available evidence. It has been suggested that this latter dimension relates to “flexible thinking,” which is viewed by Baron (2019) as being central to the concept of actively open-minded thinking. In other words, those individuals who are most able to change their beliefs, values and opinions flexibly on the basis of new evidence exemplify what it means to have an actively open-minded thinking style. Indeed, as noted by Pennycook et al. (2020), the actively open-minded thinking scale was originally created to assess, in part, people’s belief that it is good to seek evidence that may conflict with their intuitions.

In the context of tasks that induce a negative footprint illusion, it can readily be seen how useful it would be to have a thinking style whereby an intuitive “averaging” response to the carbon footprint estimation request is reflected upon more fully such that evidence is sought to determine whether a solution based on an averaging process is correct. It would be useful for future studies of the negative footprint illusion to test more directly the idea that those individuals who score highly on measures of actively open-minded thinking do indeed overturn initial intuitive responses in favor of correct responses after a period of reflective thinking. One research technique that can be deployed to explore this issue is the “two-response paradigm,” which has been established by Thompson et al. (2011) to disentangle people’s Type 1 intuitive reasoning responses from their Type 2 reflective responses (see also Thompson et al., 2013). In this paradigm, participants must first give a very fast, intuitive response and are subsequently allowed time to deliberate so that they can generate a revised response, if they wish to. In this way the consequential impact of Type 2 processing can be observed when initial, incorrect intuitive answers are replaced with correct answers after a period of reflective processing. It would, therefore, be predicted that those high in reflective reasoning dispositions, as indexed by scores on the AOT scale, would show a pattern of reasoning in which they switch from incorrect to correct responding under two-response conditions.

Studies 1 and 2 also provided evidence for a significant positive relationship between actively open-minded thinking and the CRT-*Reflective* score. Previous research has used various versions of the CRT to explore people’s propensity to overcome intuitive thinking and to engage in Type 2 reflective thinking. Such concepts are relevant to the investigation of the averaging bias that may underpin the negative footprint illusion given that this bias might derive from the application of more rapid, automatic and intuitive reasoning (i.e., Type 1 processing; Evans and Stanovich, 2013a,b; see also De Neys, 2006). In contrast, the possibility of overcoming the bias might require slower, analytic and reflective reasoning (i.e., Type 2 processing; Evans and Stanovich, 2013a,b), which can enable the reasoner to derive an appreciation of the need to produce a summative response to the task rather than an averaging response. Study 1 did not demonstrate a role for the CRT in predicting reduced susceptibility to the negative footprint illusion. However, Study 2 revealed some limited evidence for the possible association between CRT-*Reflective* scores and avoidance of the negative footprint illusion in terms of a borderline significant relationship in the context of the buildings task (*p* = 0.053) and a marginal relationship in the context of the cars task (*p* = 0.088).

The lack of a clear-cut association between performance on the CRT and reduced susceptibility to the negative footprint illusion may at first sight seem curious considering the reliable association observed between actively open-minded thinking and reduced susceptibility to the illusion. Both the CRT and the AOT scales are presumably tapping a disposition to engage in Type 2 reflective reasoning, so why is one measure less predictive than the other when it comes to assessing avoidance of the negative footprint illusion? The resolution to this issue may relate to the proposal that the actively open-minded thinking measure is a *purer* index of Type 2 thinking dispositions than CRT performance (Newton et al., 2021). This is because people can perform successfully on the CRT for reasons other than through the engagement of Type 2 reflective thinking. Indeed, there is recent evidence suggesting that some people can “intuit” the correct answers to the CRT using rapid Type 1 thinking (Raoelison et al., 2020). As such, the CRT may well be a less reliable measure of the disposition toward reflective, analytic thinking than a measure based on the use of an actively open-minded thinking scale.

In sum, our research provides evidence in support of general dispositions to engage in reflective thinking (as captured by a measure of actively open-minded thinking) in offering only limited protection against susceptibility to the negative footprint illusion. Indeed, we note caution in potentially encountering Type 1 errors when conducting multiple repeated correlations such as those presented in both Studies 1 and 2. With respect to environment-specific thinking dispositions, we found no evidence for such dispositions being associated with susceptibility to, or avoidance of, the negative footprint illusion. Indeed, the lack of evidence regarding the role of environment-specific thinking dispositions lends support to the idea that the negative footprint illusion is a general cognitive bias, potentially underpinned by the application of an “averaging” process (Holmgren et al., 2018a). We speculate that whilst avoidance of the negative footprint illusion does appear to have a relationship to dispositions to engage in reflective thinking, the amount of variance explained by measures of actively open-minded thinking remains quite low, and limited to only actively open-minded thinking. Furthermore, we also note the absence of any significant relationship between CRT-*Intuitive* and CRT-*Reflective* scores and susceptibility to the illusion in Study 1, and only borderline significant correlations between CRT-*Reflective* and susceptibility to the negative footprint illusion in the cars and buildings item types in Study 2. This indeed indicates only a limited role in terms of general dispositions to engage in reflective thinking (as captured by a measure of actively open-minded thinking) in predicting susceptibility to the negative footprint illusion. Thus, avoidance of the illusion might have a stronger association with more fundamental aspects of cognitive processing that underpin successful thinking and reasoning. To date, however, we are not aware of any studies that have explored the association between individual differences in core aspects of cognition and the negative footprint illusion, with such investigations representing a fertile avenue for further, systematic research.

Finally, we note that it remains important to determine further individual differences beyond actively open-minded thinking that might underpin susceptibility to the negative footprint illusion in a bid to determine the ways in which psychological barriers, such as cognitive bias, might be alleviated in relation to people’s environmentally oriented reasoning and decision making (Gifford, 2011). This, in turn, will hopefully shed light on practical interventions that might be implemented in real-world contexts, which will ultimately help individuals to make environmentally friendly choices (e.g., relating to the purchase of a new vehicle).

## Data Availability Statement

The raw data supporting the conclusions of this article will be made available by the authors, without undue reservation.

## Ethics Statement

The studies involving human participants were reviewed and approved by University of Gävle, Gävle, Sweden and University of Central Lancashire, Preston, United Kingdom. The patients/participants provided their written informed consent to participate in this study.

## Author Contributions

ET, JM, MH, HA, and LB contributed to the conception and design of the studies. ET, JM, and MN conducted the data coding and analysis. ET wrote the first draft of the manuscript with advice from JM. LB and JM worked on the second draft. LB wrote sections of the manuscript. All authors contributed to the article and approved the submitted version.

## Conflict of Interest

The authors declare that the research was conducted in the absence of any commercial or financial relationships that could be construed as a potential conflict of interest.

## Publisher’s Note

All claims expressed in this article are solely those of the authors and do not necessarily represent those of their affiliated organizations, or those of the publisher, the editors and the reviewers. Any product that may be evaluated in this article, or claim that may be made by its manufacturer, is not guaranteed or endorsed by the publisher.
