# Supplementary material for: Biased Estimates of Environmental Impact in the Negative Footprint Illusion: The Nature of Individual Variation
Source: Front Psychol. 2022 Jan 18;12:648328. doi: 10.3389/fpsyg.2021.648328 (PMC8803658; doi:10.3389/fpsyg.2021.648328)
Supplement: Supplementary file 1 [file Table_1.DOCX]

**Supplementary Materials**

**Study 1**

***Carbon Footprint Estimation: Rating the Carbon Footprint of Apples***

***First Rating:*** Participants were presented with instructions, contextual information relating to a carbon footprint and an anchor-point, as follows: *“Below is a picture of apples, which represent a number of apples in a food basket. Your task is to estimate how large the carbon footprint is for these apples.”*

*Carbon footprint refers to the carbon dioxide emissions that are released from, for example, transportation and farming. A high carbon footprint is therefore* ***worse*** *for the environment, whereas a low carbon footprint is* ***better*** *for the environment. A reference point you can have in mind while making your estimate is that a bowl of mixed fruit consisting of 10 different fruits would have scored five on the scale.”*

Participants rated the carbon footprint of the 10 apples in the consumers basket on a scale from 1 (low carbon footprint) to 9 (high carbon footprint): “*These apples represent 10 conventional apples in a consumers food basket.* [please refer to picture 1a]. *Please estimate the carbon footprint for the 10 apples in the consumers food basket”.*

***Second Rating:*** Following the initial rating, participants were presented with further contextual information, this time relating to the concept of environmentally friendly items: *“Environmentally friendly or environment-friendly (also referred to as eco-friendly, nature-friendly and green) are sustainability and marketing terms referring to goods and services, laws, guidelines and policies that claim minimal harm upon eco-systems or the environment. Below is a picture of apples which represent a number of apples in a food basket. Your task is to estimate how large the carbon footprint is for these apples.”*

*“Carbon footprint refers to the carbon dioxide emissions that are released from, for example, transportation and farming. A high carbon footprint is therefore* ***worse*** *for the environment, whereas a low carbon footprint is* ***better*** *for the environment*. *You will make your estimate on a scale from 1 (low carbon footprint) to 9 (high carbon footprint).”*

Participants rated the carbon footprint of the 10 apples in the consumers basket on a scale from 1 (low carbon footprint) to 9 (high carbon footprint): *“These apples represent 10 conventional apples in a consumer's food basket. The consumer went back into the store and added 5 eco-friendly apples to their food basket.*[please refer to picture 1b]. *Please estimate the carbon footprint for the 15 apples in the consumers food basket.”*

***Carbon Footprint Estimation: Rating the Carbon Footprint of Buildings***

***First Rating:*** Participants were asked to provide a rating for the number of trees required to compensate for the carbon footprint arising from the energy used by a community of buildings (i.e., houses). Participants were presented with the following contextual information, alongside a graphic of 75 conventional buildings marked in orange, and a depiction of the current and potential energy efficiency rating for each building in the standard A to G colour-coded scheme: *“A suburb consisting of 75 conventional buildings was built in 1950 by Boverket (national board of housing, building and planning) and comprises apartments, villas and semi-detached houses. All units have the same materials and performance characteristics. Number of Units: 75 Conventional Units.”* [see picture 1c]. “*Note: Current refers to the actual building energy efficiency. Potential refers to the possible rating after improvements.”*

Participants were also presented with information relating to greenhouse gas emissions: *“Energy use is linked to greenhouse gas (GHC) emissions, which in turn are harmful for the environment. For example, assume for every unit of electrical energy used and for every unit of residential heat used, a certain amount of CO_2_ is produced. A transportation company based in Orebro, has introduced an environment compensation policy, that is, if one of their cars covers 33,000 Km, the company will plant 50 trees. Planted trees absorb CO_2_ for many years and can therefore compensate for the negative environmental consequences of increased energy use.”*

Participants were asked to mark the number of trees used to compensate for energy use on a scale from 1 to 100. “*Based on this understanding, please mark on the scale below how many trees you estimate the suburb will need to compensate for its energy use per month”.*

***Second Rating:*** Following the initial rating, participants were presented with the same contextual information as previously: “*A suburb consisting of 75 conventional buildings was built in 1950 by Boverket (national board of housing, building and planning) and comprises of apartments, villas and semi-detached houses. All units have the same materials and performance characteristics.”* The following information was also added: *“In 2014, 25 units of green buildings were built to address the housing shortage. All new units have the same materials and performance*. *Number of units: 75 Conventional Units + 25 Green Buildings.* [see picture 1d]. *Note: Current refers to the actual building energy efficiency. Potential refers to the possible rating after improvements.”* This information was presented alongside a graphic depicting 75 conventional buildings in orange together with the 25 new eco-friendly buildings in green.

Participants were then presented with the same information as was presented with the initial rating: *“Energy use is linked to greenhouse gas (GHC) emissions, which in turn are harmful for the environment. For example, assume for every unit of electrical energy used and for every unit of residential heat used, a certain amount of CO_2_ is produced. A transportation company based in Orebro, has introduced an environment compensation policy, that is, if one of their cars covers 33,000 Km, the company will plant 50 trees. Planted trees absorb CO_2_ for many years and can therefore compensate for the negative environmental consequences of increased energy use.”*

Participants were asked once again to mark on the scale from 1 to 100 how many trees they estimated the suburb would need to compensate for its energy use per month: *“Based on this understanding, please mark on the scale below how many trees you estimate the suburb will need to compensate for its energy use per month”.*

***General Dispositional Measures of Reasoning***

***The Cognitive Reflection Test (CRT):*** The 6-item version contains three items originally used by Frederick (2005), as follows:

1. *A bat and ball costs £1.10 in total. The bat costs £1.00 more than the ball. How does the ball cost?*
2. *It takes 5 machines 5 minutes to make 5 widgets, how long would it take 100 machines to make 100 widgets?*
3. *In a lake, there is a patch of lily pads. Every day, the patch doubles in size. If it takes 48 days for the patch to cover the entire lake, how long would it take for the patch to cover half the lake?*

Primi et al. (2016) added a further three items to create the CRT-L, as follows:

4. *If three elves can wrap three toys in 1 hour, how many elves are needed to wrap six toys in 2 hours?*

*5. Jerry received both the 15th highest and the 15th lowest mark in the class. How many students are there in the class?*

6. *In an athletics team, tall members are three times more likely to win a medal than short members. This year the team has won 60 medals so far. How many of these have been won by short athletes?*

Primi et al. (2016) applied item response theory to investigate the psychometric properties of the CRT-L and found that these properties were good and superior to those of the original 3-item CRT, supporting the use of the CRT-L in studies of reasoning.

The CRT-L was scored according to the process outlined by Pennycook et al. (2016). The correct items were totaled, and a mean proportion calculated, to provide a CRT-*Reflective* score. This score measures the ability of individuals to overcome intuitive responses and reach a normatively correct response. A corresponding CRT-*Intuitive* score can be derived, which represents a measure of solutions that spring to mind rapidly and seem plausible, but which are incorrect. The CRT-*Intuitive* score involves totaling these intuitive but incorrect responses and converting the total into a mean proportion.

***Other Measures***

***Susceptibility to the Conjunction Fallacy*:** The tasks were the famous “Linda Problem” and a less well known “Die-Roll Problem”, as follows:

1. *Linda is 31 years old, single, outspoken, and very bright. She majored in philosophy. As a student, she was deeply concerned with issues of discrimination and social justice, and also participated in anti-nuclear demonstrations. Which is more probable: (i) Linda is a bank teller; or (ii) Linda is a bank teller and is active in the feminist movement?*

2. *Consider a regular six-sided die with four green faces and two red faces.  The die will be rolled 20 times and the sequences of greens (G) and reds (R) will be recorded.  You are asked to select one sequence, from a set of three, and you will win £25 if the sequence you chose appears on successive rolls of the die.* *Please check the sequence of greens and reds on which you prefer to bet: (i) RGRRR; (ii) GRGRRR; or (iii) GRRRRR.*

To be scored as correct on these tasks the participant needed to endorse the probability of a single event happening (e.g., that Linda is a bank teller) as being more likely than the probability of two events happening in conjunction (e.g., that Linda is a bank teller and is active in the feminist movement).

***Intuition vs. Analysis Response Instructions***

The following instructions were provided for an intuition response: *“Intuition means that the answer suddenly came to your mind while you were trying to solve the problem, even though you are unable to articulate how you achieved the solution. This kind of solution is associated with a lack of conscious deliberation and little effort”.*

The following instructions were provided for an analysis response: *“Analysis means that you figured out the answer after you deliberately and consciously tested out different ideas before you found the right one. In this case for instance, you are able to report the steps that you used to reach the solution”*

**Study 2**

***Carbon Footprint Estimation: Rating the Carbon Footprint of Apples***

***First Rating:*** This task retained some features of that presented in Study 1, but changes were made to the contextual information and the response scale. Participants were presented with the following contextual information: *“A consumer’s food basket contains 10 apples. There are 10 apples pictured below.*[please see picture 1a].

*“Your task is to estimate the carbon footprint of the 10 apples in the consumer’s food basket. Carbon footprint refers to the carbon dioxide emissions that are released as a result of, for example, transportation. A high carbon footprint is therefore worse for the environment whereas a low carbon footprint is better for the environment.”*

Participants were asked to estimate the carbon footprint of 10 apples on a scale from “low carbon footprint” (‘1’) to “high carbon footprint” (‘100’). “*As a reference point for your estimations, consider that a basket of 10 mixed fruit would score in the middle of the scale. Please respond by moving the slider to provide your answer.”*

***Second Rating:*** Following the initial rating, participants were presented with further contextual information, this time related to the concept of environmentally friendly items: *“There are 10 apples in a consumer’s food basket, and the consumer decided to add a further 5 green (eco-friendly) apples to their basket. Eco-friendly apples have a low environmental impact and are produced using methods that reduce their carbon footprint and lowers the energy required in their production. There are 10 apples and 5 green (eco-friendly) apples pictured below.”* [please see picture 1b]

Participants were then required to estimate the combined carbon footprint of the 10 apples and the 5 green (eco-friendly) apples pictured on a scale from “low carbon footprint” (‘1’) to “high carbon footprint” (‘100’). *“How large a carbon footprint (impact on the environment) do you estimate that the 15 apples in the consumer’s food basket have together? Please respond by moving the slider to provide your answer.”*

***Carbon Footprint Estimation: Rating the Carbon Footprint of Cars***

***First Rating:*** Participants were asked to rate the carbon footprint of a fleet of cars. They were presented with instructions, contextual information relating to a carbon footprint and an anchor-point, as follows: “*A company owns a fleet of 30 conventional petrol cars. There are 30 blue (conventional) petrol cars pictured below.”* [please see picture 2c]

“*Your task is to estimate the carbon footprint of the 30 conventional petrol cars in the fleet. Carbon footprint refers to the carbon dioxide emissions that are released as a result of, for example, engine size. A high carbon footprint is therefore worse for the environment whereas a low carbon footprint is better for the environment.”*

Participants were asked to estimate the carbon footprint of 30 petrol cars on a scale from “low carbon footprint” (‘1’) to “high carbon footprint” (‘100’). *As a reference point for your estimations, consider that a fleet of 30 motorcycles would score in the middle of the scale. Please respond by moving the slider to provide your answer”.*

***Second Rating:*** Participants were presented with further contextual information, this time related to the concept of environmentally friendly items: *“There are 30 blue (conventional) petrol cars in a fleet, and the owners of the company decide to add a further 15 green (eco-friendly) electric cars to the fleet. Eco-friendly cars have a low environmental impact and are designed and built using materials and technology that reduces their carbon footprint and lowers their energy requirements. There are 30 blue (conventional) petrol cars and 15 green (eco-friendly) electric cars pictured below.”* [see picture 2d]

Participants were then required to estimate the carbon footprint of the 30 blue (conventional) petrol cars and the 15 green (eco-friendly) electric cars pictured, on a scale from “low carbon footprint” (‘1’) to “high carbon footprint” (‘100’). “*How large a carbon footprint (impact on the environment) do you estimate that the 45 cars in the fleet have together? Please respond by moving the slider to provide your answer.”*

***Carbon Footprint Estimation: Rating the Carbon Footprint of Buildings***

***First Rating:*** Participants were asked to rate the carbon footprint of a community of houses. A small number of changes were made to the corresponding estimation task from Study 1. Participants were provided with the following contextual information: *“A community consists of 50 orange (conventional) houses. There are 50 orange (conventional) houses pictured below.”* [see picture 2e]

*“Your task is to estimate the carbon footprint of the 50 orange houses in the community. Carbon footprint refers to the carbon dioxide emissions that are released, as a result of, for example, powering appliances. A high carbon footprint is therefore worse for the environment, whereas a low carbon footprint is better for the environment.”*

Participants were asked to estimate the carbon footprint of 50 conventional houses on a scale from “low carbon footprint” (‘1’) to “high carbon footprint” (‘100’). *As a reference point for your estimations, consider that a community of 50 apartments would score in the middle of the scale. Please respond by moving the slider to provide your answer”.*

***Second Rating:*** Participants were presented with further contextual information, this time related to the concept of environmentally friendly items: *“There are 50 orange (conventional) houses in a community, and the builders decide to add a further 25 green (eco-friendly) houses to the community. Eco-friendly houses have low environmental impact and are designed and built using materials and technology that reduces their carbon footprint and lowers their energy requirements. There are 50 orange (conventional) houses and 25 green (eco-friendly) houses pictured below.”* [please see picture 2f]

Participants were then required to estimate the carbon footprint of the 50 conventional houses and the 25 green (eco-friendly) houses, on a scale from “low carbon footprint” (‘1’) to “high carbon footprint” (‘100’). “*How large a carbon footprint (impact on the environment) do you estimate that the 75 houses in the community have together? Please respond by moving the slider to provide your answer.”*

**Carbon Footprint Estimation Tasks**

**Apples – Study 1 & Study 2**

**Picture 1a**


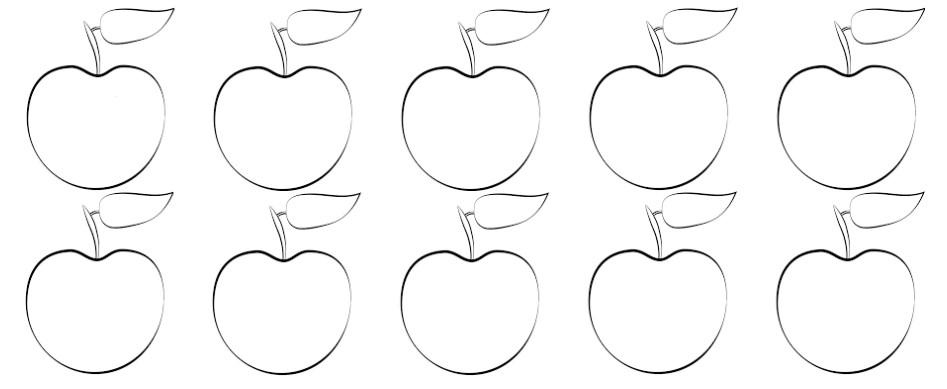


**Picture 1b**

**
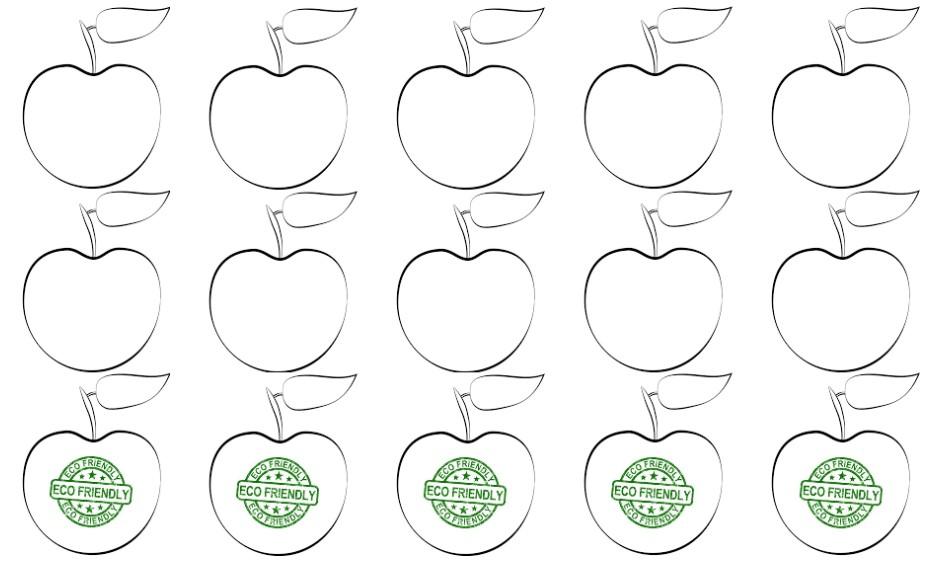
**

**Buildings – Study 1**

**Picture 1c**

**
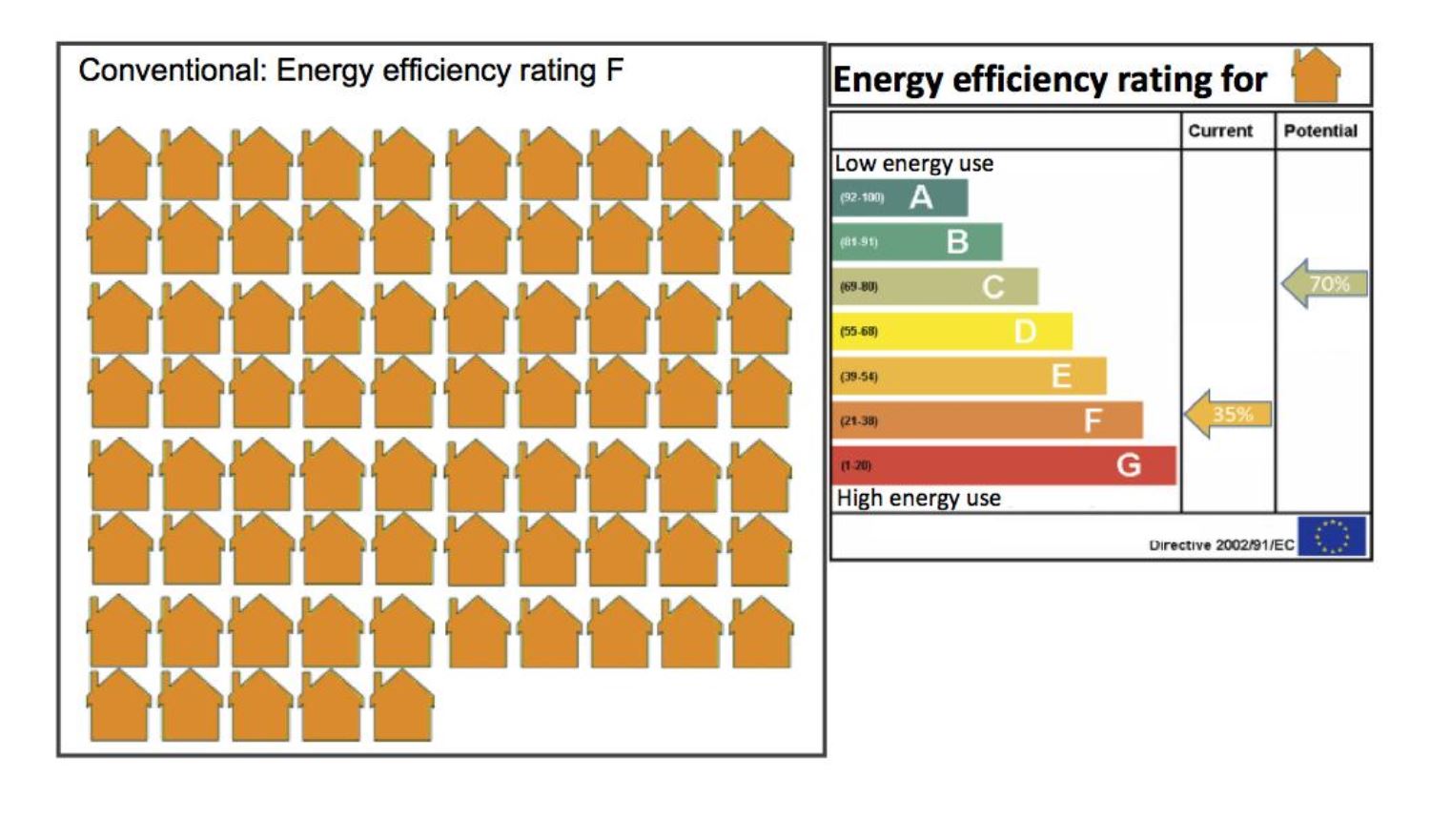
**

**Picture 1d**

**
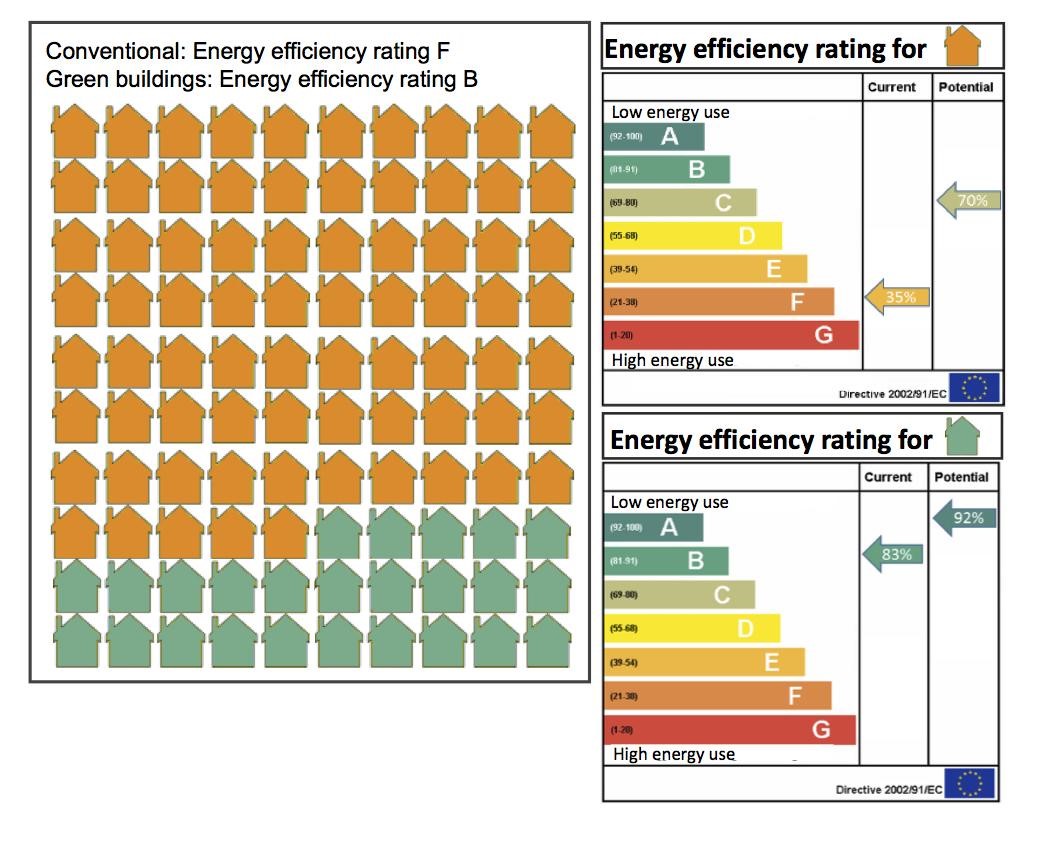
**

**Cars – Study 2**

**Picture 2c**

**
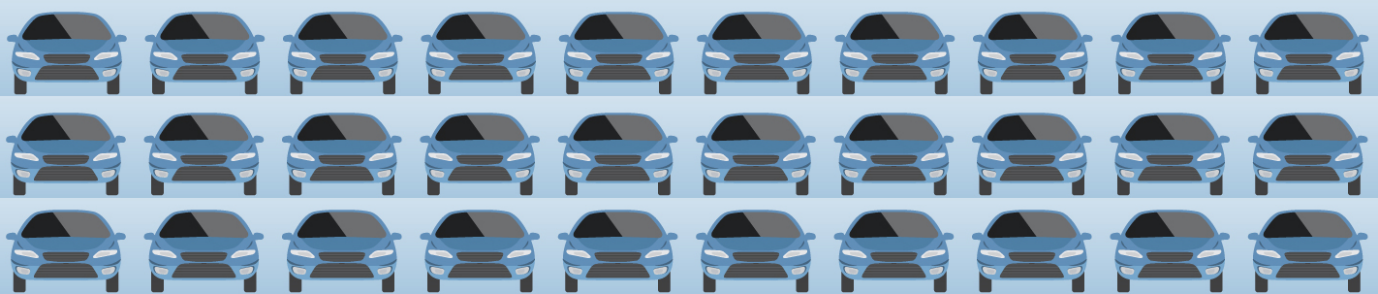
**

**Picture 2d**

**
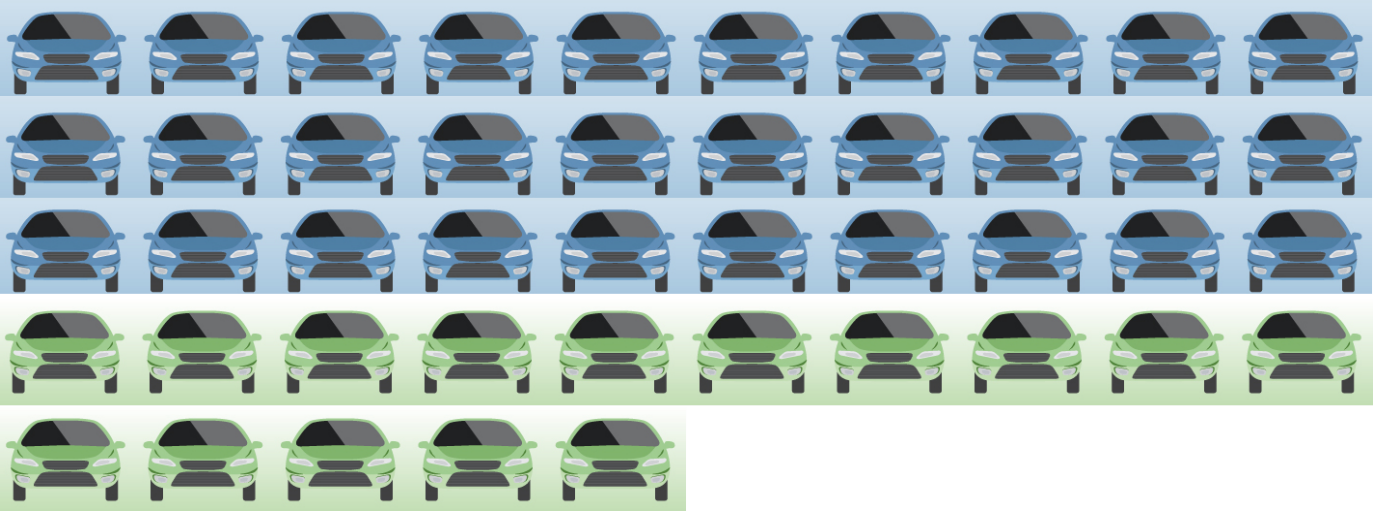
**

**Buildings – Study 2**

**Picture 2e**

**
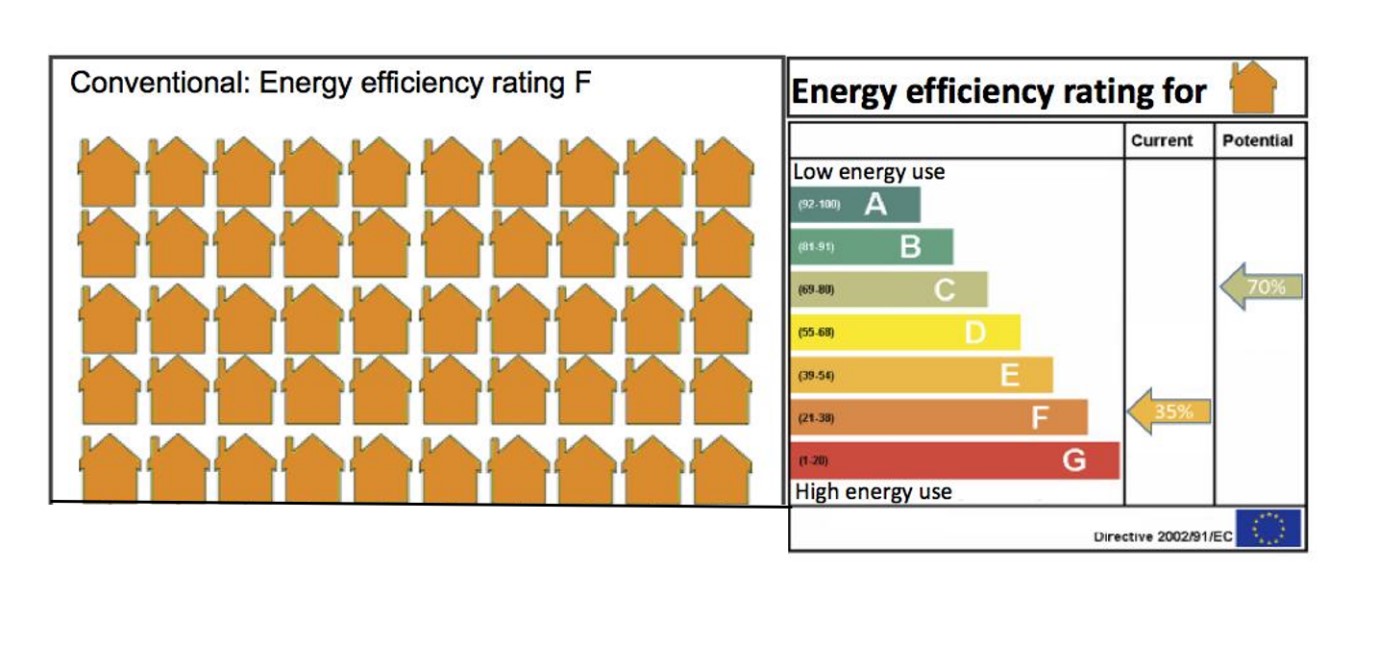
**

**Picture 2f**

**
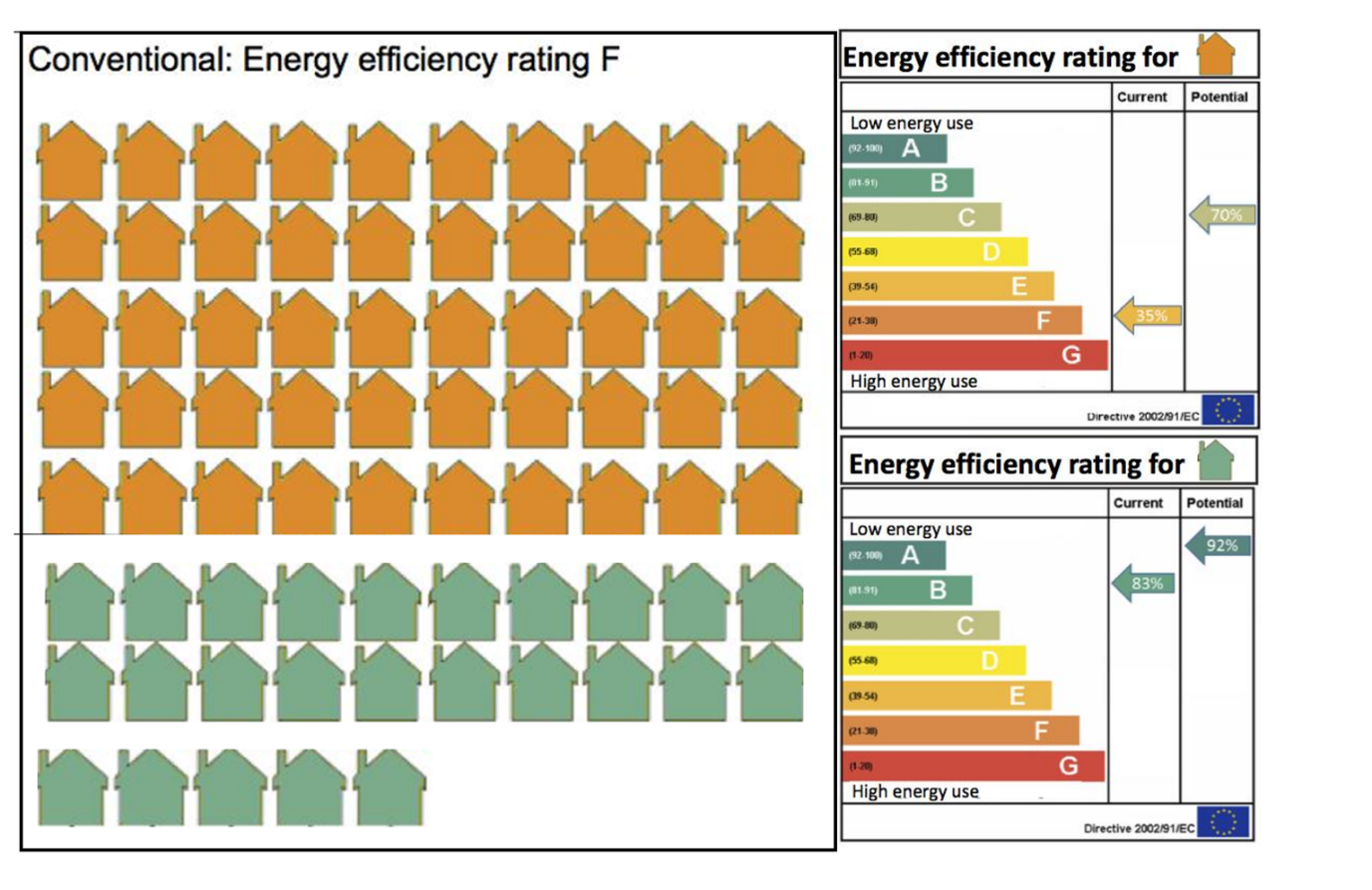
**
